# Hyperbranched polydendrons: a new nanomaterials platform with tuneable permeation through model gut epithelium†
†Electronic supplementary information (ESI) available: Materials, full experimental details and characterisation. See DOI: 10.1039/c4sc02889a



**DOI:** 10.1039/c4sc02889a

**Published:** 2014-10-03

**Authors:** Fiona L. Hatton, Lee M. Tatham, Louise R. Tidbury, Pierre Chambon, Tao He, Andrew Owen, Steven P. Rannard

**Affiliations:** a Department of Chemistry , University of Liverpool , Crown Street , L69 7ZD , UK . Email: srannard@liv.ac.uk; b Department of Molecular and Clinical Pharmacology , University of Liverpool , Block H, 70 Pembroke Place , Liverpool L69 3GF , UK; c Institute of Chemical and Engineering Sciences , Agency for Science , Technology and Research (A*STAR) , 1, Pesek Road, Jurong Island , 627833 , Singapore

## Abstract

The development of nanomaterials for advanced therapies requires the formation of versatile platforms that may be tuned to maximize beneficial attributes and minimize unwanted negative behaviour. Additionally, the optimum route of administration is a key consideration of any new treatment and much work has been focused on direct injection into the systemic circulation rather than oral delivery. Here we describe a new approach to polymeric nanoparticle design and present initial results showing the potential for tuneable permeation through a gut epithelium model. Through the use of mixed initiators and branched vinyl polymerization, a series of systematically varying branched polymers have been synthesized and nanoprecipitated. The surprisingly uniform structures have undergone preliminary pharmacological evaluation to establish low cytotoxicity and enhanced permeation through model intestinal epithelial cells. This presents potential opportunities for future developments that may allow oral dosing to result in circulating polymeric nanoparticles; behaviour that may prove clinically desirable to many non-terminal or chronic diseases that utilise nanomedicines but wish to avoid regular or repeated intravenous administration.

## Abstract 2

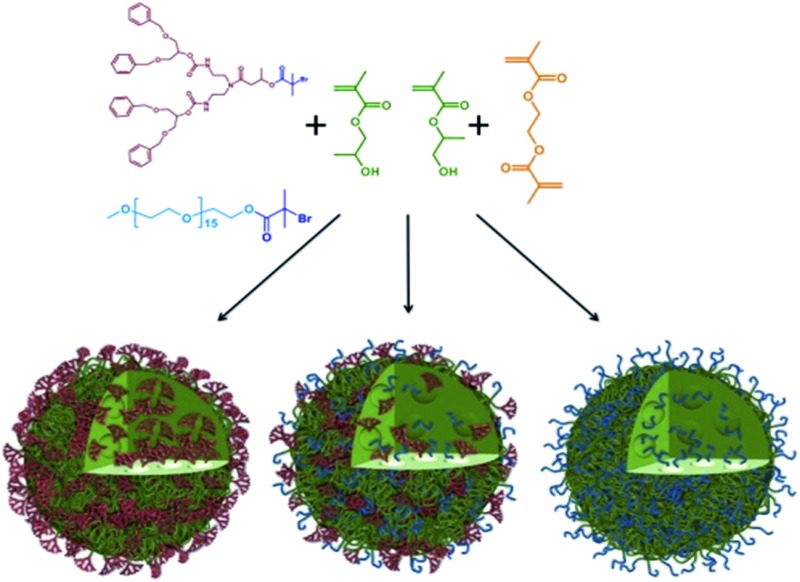
Highly branched vinyl polymers (hyperbranched polydendrons), displaying combinations of dendritic and PEG end groups, have been synthesised using a mixed initiator approach. Nanoprecipitated polydendron particles have exhibited controlled permeation through a gut epithelium model.

## Introduction

Clinically successful nanomedicines predominantly utilize two general approaches: orally-dosed solid-drug nanoparticles (SDNs)^1–3^ and intravenous drug-nanocarriers.^4–6^ Oral dosing is globally the most patient-acceptable drug administration format, often requiring no intervention by trained healthcare workers, offering reduced costs and requiring lower levels of sterility.^7,8^ SDN strategies aim predominantly to enhance the low oral bioavailability observed with many drug classes which may force large clinically administered doses or lead to the potential failure of new drug candidates. Relatively recently, SDNs have also been clinically evaluated as depot injections to provide long-term (months) exposure from a single subcutaneous or intramuscular injection of particulate drugs.^9–11^ Injected drug-nanocarriers (sometimes described as drug delivery systems) are typically administered intravenously and offer enhanced systemic circulation, lower doses, reduction of side effects, and targeting/accumulation within cellular or tissue sites. Such benefits from many nanocarriers require the nanocarrier/drug vehicle to reach the systemic circulation^12^ as an intact nanoparticle whereas overcoming low bioavailability *via* SDNs may result solely from enhanced drug dissolution prior to, or during, permeation of the intestinal epithelium.^1^ Injections are not widely used clinically for chronic or non-terminal diseases and a clear, yet highly challenging, target for nanomedicine is the development of orally-dosed nanocarriers that can deliver drugs into the systemic circulation whilst maintaining their particulate form. Candidates such as polymeric micelles, polymer-drug conjugates, nanoprecipitates and nanoemulsions are undergoing considerable study in this respect,^13–16^ however, the synthetic/biological challenge and difficulties of *in vivo* detection within the blood, and during clearance, continue to act as a barrier to translation.

Arguably the most diverse organic nanomaterial studies have involved ideally-branched dendrimers,^17–19^ and linear–dendritic polymer hybrids.^20^ Multi-step convergent or divergent dendrimer syntheses have led to branched polymer chemistries, from polyimines/polyamines^21^ and polycarbonates^22^ through to polyamides,^23,24^ polyamido-amines,^25^ polyesters,^26^ polyurethanes,^27^ and polycarbosilanes^28^ to name a selection, and dendritic polymer application^29^ has begun to mature in nanomedicine.^30^ Obstacles continue to exist for cost-effective applications including the synthetic complexity and molecular weight/physical size limitations. Although dendrimers possess uniform molecular weight distributions and well defined shape, the available size range has long been limited to <15 nm^31^ until very recent reports of generation 13 triazine-derived materials with molecular weights >8 × 10^6^ g mol^–1^ and diameters of 30 nm.^32^

Many dendrimer materials also require lengthy syntheses that often utilize large reagent excesses. To provide broader materials options, dendrimer sub-units (dendrons) have been attached to linear polymer chain-ends or side-chains to form linear–dendritic hybrid block copolymers with multiple additional functionality and supramolecular assembly properties.^20^ Micellar structures of amphiphilic linear–dendritic copolymers (30–70 nm),^26^ and micelle aggregates (approx. 200 nm),^33^ have been reported after association of relatively small building blocks. The control of structures within the 30–250 nm size range is of interest in drug delivery (conjugated or encapsulated drug),^19^ with success reported for combinations of dendrons with linear polymers leading to greater flexibility and scope for molecular design and the direction of particle formation.

One-pot, branched vinyl copolymerization techniques^34^ have been shown to form nanoparticles in a single synthetic procedure^35^ *via* a concerted propagation and interchain branching of linear polymer chains using low concentrations of bifunctional monomers. When used in conjunction with controlled radical polymerization techniques, high molecular weight branched amphiphilic block copolymers have been formed that produce spherical nanoparticles when dialysed using water.^36^ The introduction of multi-functional initiators have also allowed dumbbell and clover-leaf nanoparticles to be formed,^37^ however, the loss of chain end functional multiplicity distances these materials from ideal dendrimers or linear–dendritic hybrids.

Herein we combine the aspects of linear–dendritic hybrids and branched vinyl polymerization to provide high molecular weight dendron-functional macromolecules, termed hyperbranched-polydendrons (hyp-polydendrons).^38^ By utilizing mixed initiator systems, this new synthetic strategy allows a ready methodology to systematically control mixed surface functionality and form complex spherical nanoparticles. These materials have: (1) a unique control of surface functionality; (2) directed aqueous self-assembly; (3) low dispersity when used to form nanoparticles with controlled diameters (75–210 nm); (4) encapsulation capabilities; and (5) variable interaction with model gut epithelial monolayers. Our preliminary studies of interactions of hyp-polydendron nanoprecipitates with a gut epithelium model show potential value in further work to understand and optimize nanoparticle transport across the gut barrier and the benefits that might be achievable after oral dosing.

## Results and discussion

### Synthesis of hyp-polydendrons with mixed functionality

**Fig. 1:**
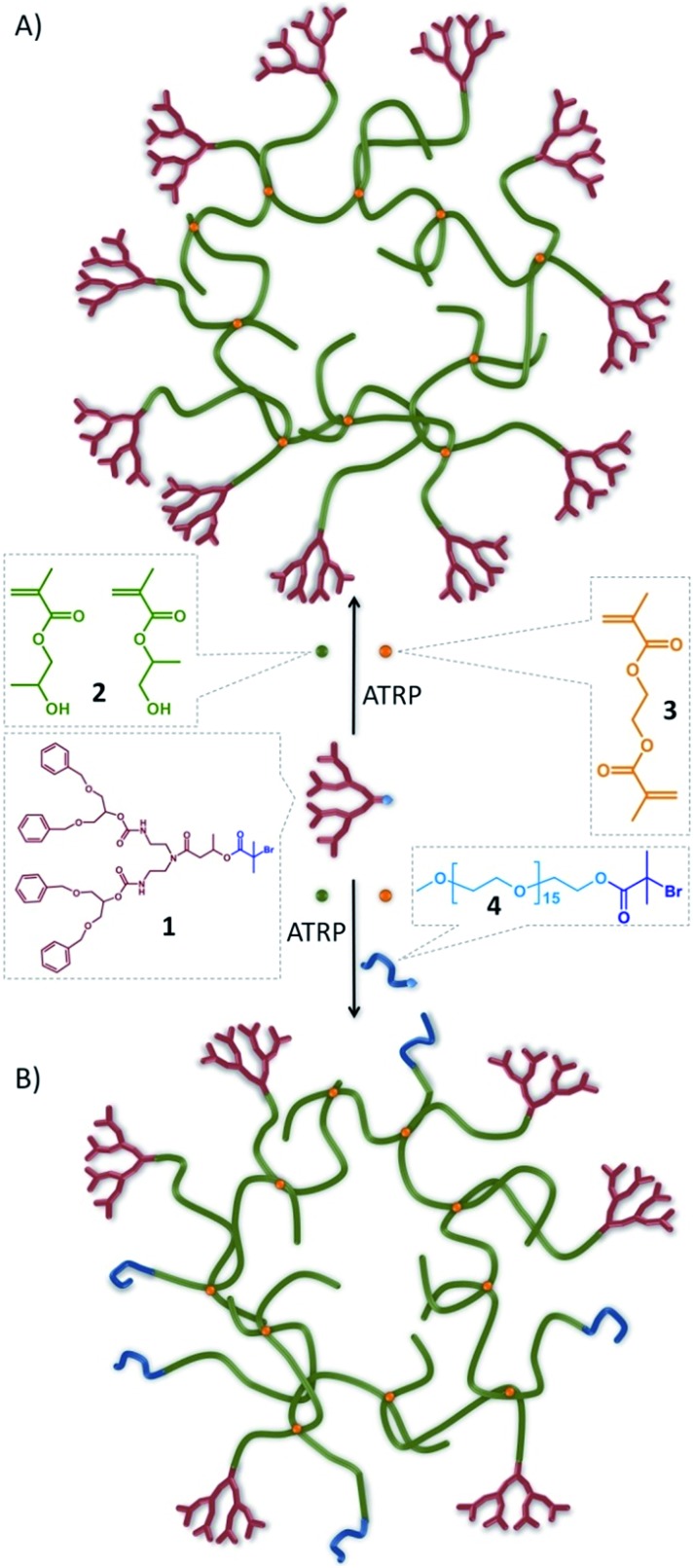
Schematic representation of idealised hyp-polydendron synthesis. Atom transfer radical copolymerization (ATRP) of 2-hydroxypropyl methacrylate, **2**, and ethylene glycol dimethacrylate, **3**, initiated by the functional G_2_ dendron, **1** to form (A) hyp-polydendrons; and (B) inclusion of a PEG_16_-initiator, **4**, to form hyp-polydendrons with controlled and mixed surface functionality.

To collectively overcome the synthetic complexity of dendrimer synthesis, and readily provide materials with the functional benefits of dendrimers, we recently reported the use of a dendron-derived initiator, **1**, in the copper catalysed, branched vinyl atom transfer radical (ATRP) copolymerization of 2-hydroxypropyl methacrylate (HPMA), **2**, and ethylene glycol dimethacrylate (EGDMA), **3**, to form novel polymer architectures containing large numbers of dendrons at one end of each conjoined primary polymer chain^38^ (Fig. 1A). We have termed these materials hyp-polydendrons as they combine a highly branched vinyl polymer core with dendron-derived chain ends to form new hyperbranched linear–dendritic hybrid architectures.

In the absence of **3**, dendron-initiated methanolic ATRP polymerizations of **2** (target number average degree of polymerization DP_n_ = 50 monomer units), using the second generation (G_2_) dendron initiator, **1**, led to high monomer conversion (>99%) and recovered polymers with a number average molecular weight (*M*_n_) of 12 200 g mol^–1^ and weight average molecular weight (*M*_w_) of 16 600 g mol^–1^, as determined by triple detection size exclusion chromatography (SEC; Table 1; Fig. S1 & S2†). In the presence of **3** (**1** : **3** molar ratio of 1 : 0.8), polymerizations also reached high conversion (>99%) with no observable interference of the controlled radical polymerization kinetics (Fig. S3†), but polymers of considerably higher molecular weight (*M*_n_ = 90 500 g mol^–1^; *M*_w_ = 1 304 000 g mol^–1^) were recovered (Table 1).

**Table 1:** SEC characterisation of polydendrons produced using varying G_2_ dendron–PEG_16_ initiator ratios and under varying polymerization conditions

Initiator (mol fraction)		Target polymer composition	SEC		
G_2_	PEG_16_		*M*_n_	*M*_w_	*Đ* (*M*_w_/*M*_n_)
1	0	HPMA_50_ ^*a*^	12 200	16 600	1.36
0.5	0.5	HPMA_50_ ^*a*^	13 000	16 900	1.30
0	1	HPMA_50_ ^*a*^	10 600	16 800	1.59
1	0	HPMA_50_–EGDMA_0.8_ ^*b*^	90 500	1 304 000	14.40
0.9	0.1	HPMA_50_–EGDMA_0.8_ ^*b*^	68 500	1 495 000	21.84
0.75	0.25	HPMA_50_–EGDMA_0.8_ ^*b*^	52 400	987 800	18.88
0.5	0.5	HPMA_50_–EGDMA_0.8_ ^*b*^	39 400	480 700	12.19
0.25	0.75	HPMA_50_–EGDMA_0.8_ ^*b*^	36 200	315 300	8.73
0.1	0.9	HPMA_50_–EGDMA_0.8_ ^*b*^	37 700	286 000	7.61
0	1	HPMA_50_–EGDMA_0.8_ ^*b*^	68 100	296 200	4.35
0.5	0.5	HPMA_50_–EGDMA_0.95_ ^*c*^	121 900	1 779 000	14.60
0	1	HPMA_50_–EGDMA_0.95_ ^*c*^	74 700	642 700	8.60

^*a*^Linear polymerization.

^*b*^Branched polymerizations at total initiator : brancher ratio = 1 : 0.8.

^*c*^Branched polymerizations at total initiator : brancher ratio = 1 : 0.95.

Dendrimers with statistically mixed (divergent synthesis) and controlled mixing (convergent synthesis) of surface functionality have been reported previously.^39–44^ The hyp-polydendron synthesis strategy offers a unique opportunity for systematic incorporation of mixed functionalities through the mixing of ATRP initiators. Dendron-derived initiators guarantee one dendron on each primary polymer chain, but the introduction of varying ratios of a polyethylene glycol-derived (PEG_16_) ATRP initiator, **4**, to **1** prior to copolymerization of **2** and **3** (Fig. 1B) has been undertaken. A range of branched polymerizations were conducted with **1** : **4** ratios of 100 : 0, 90 : 10, 75 : 25, 50 : 50, 25 : 75, 10 : 90 and 0 : 100, whilst maintaining the overall initiating species (based on tertiary bromide functionality) to brancher, (**1** + **4**) : **3**, ratio of 1 : 0.8. Polymerization kinetic studies confirmed controlled radical polymerization, however, the achieved molecular weights (*M*_n_ and *M*_w_) decreased with increasing content of **4**. This is quite possibly due to an overall increase in initiation efficiency generating a decrease in the effective initiator : brancher ratio and subsequent lower branching; *M*_w_ values ranging between 1 495 000–296 000 g mol^–1^ were observed across the range of initiator mixtures when utilising a (**1** + **4**) : **3** molar ratio of 1 : 0.8 (Table 1; Fig. S2†). Increased levels of **3** were tolerated within the polymerizations containing high concentrations of, **4** without the presence of gelation and *M*_w_ values between 2-fold and 4-fold higher were achievable at (**1** + **4**) : **3** molar ratio of 1 : 0.95 (Table 1); *M*_n_ values were also considerably increased.

Confirmation of the varying **1** : **4** ratio within the various hyp-polydendron structures was obtained by ^1^H nuclear magnetic resonance (NMR) spectroscopy (Fig. S4†). The calculated molar variation of G_2_ dendron and PEG_16_ end-groups were observed to follow theoretical concentrations closely (Fig. S5†); however, at higher target G_2_ dendron values, slightly lower dendron incorporation was observed, again a potential effect of the differing initiator efficiencies.

### Aqueous nanoprecipitation and characterisation of hyp-polydendrons with mixed surface functionality

Nanoprecipitation of linear polymers^45^ has previously generated uniform spherical nanoparticles predominantly from biodegradable polymers.^46^ The nanoprecipitation mechanism is proposed to undergo a nucleation/aggregation process^47^ and a multitude of factors may be varied (solvent choice, polymer concentration, viscosity) to direct the formation of colloidally stable particles of varying size.^48^ We recently demonstrated the successful aqueous nanoprecipitation of branched polymer architectures^49^ and the self-assembly of hyp-polydendrons^38^ within organic solvent mixtures and water to form uniform, spherical, self-assembled nanostructures.

Successful nanocarrier candidates (100–200 nm), formed by careful control of polymer structure and nanoprecipitation,^50,51^ have previously shown the potential for relatively high drug loadings. Chain-end modification of polyethylene glycol segments within amphiphilic A–B block copolymers by aptamers (Accurin™ technology) has also led to positive phase I evaluation of drug delivery to sites of prostate and lung cancer^51,52^ from injected nanoprecipitates with recently announced progression to phase II human studies. The seven branched polymers (one hyp-polydendron, five hyp-polydendrons with varying PEG_16_ content and one entirely PEG_16_ initiated hyp-polymer) were subjected to nanopreciptation^46,49^ under varying conditions (Table 2). A simple dropping method was employed using two initial polymer concentrations (*C*_I_) in a good solvent (10 or 5 mg mL^–1^ in tetrahydrofuran (THF)) and two dilution ranges (5 or 100-fold) into an antisolvent (water).

**Table 2:** Effect of solution and dilution concentrations on hyp-polydendron nanoprecipitation with varying G_2_–PEG_16_ molar ratios

G_2_–PEG_16_ molar ratio	Initial THF concentration (*C*_I_; mg mL^–1^)	Final H_2_O concentration (*C*_F_; mg mL^–1^)	*D*_*z*_ ^*a*^ (nm)	PdI^*a*^	*ζ* (mV)
100 : 0	10	2	106	0.083	–38.0
100 : 0	10	0.1	134	0.064	–34.3
100 : 0	5	1	81	0.083	–38.2
100 : 0	5	0.05	93	0.071	–20.4
90 : 10	10	2	173	0.076	–30.4
90 : 10	10	0.1	205	0.087	–33.1
90 : 10	5	1	116	0.069	–25.9
90 : 10	5	0.05	110	0.087	–17.6
75 : 25	10	2	155	0.085	–25.6
75 : 25	10	0.1	190	0.097	–39.0
75 : 25	5	1	110	0.073	–26.5
75 : 25	5	0.05	135	0.092	–30.2
50 : 50	10	2	148	0.061	–29.3
50 : 50	10	0.1	150	0.073	–29.6
50 : 50	5	1	115	0.067	–28.2
50 : 50	5	0.05	104	0.072	–20.7
25 : 75	10	2	121	0.072	–28.7
25 : 75	10	0.1	110	0.104	–24.0
25 : 75	5	1	93	0.078	–30.4
25 : 75	5	0.05	77	0.140	–16.5
10 : 90	10	2	129	0.058	–31.3
10 : 90	10	0.1	108	0.074	–23.2
10 : 90	5	1	94	0.091	–29.9
10 : 90	5	0.05	78	0.090	–28.9
0 : 100	10	2	121	0.074	–39.8
0 : 100	10	0.1	116	0.072	–30.0
0 : 100	5	1	90	0.083	–39.7
0 : 100	5	0.05	89	0.092	–27.8

^*a*^Measured by dynamic light scattering.

**Fig. 2:**
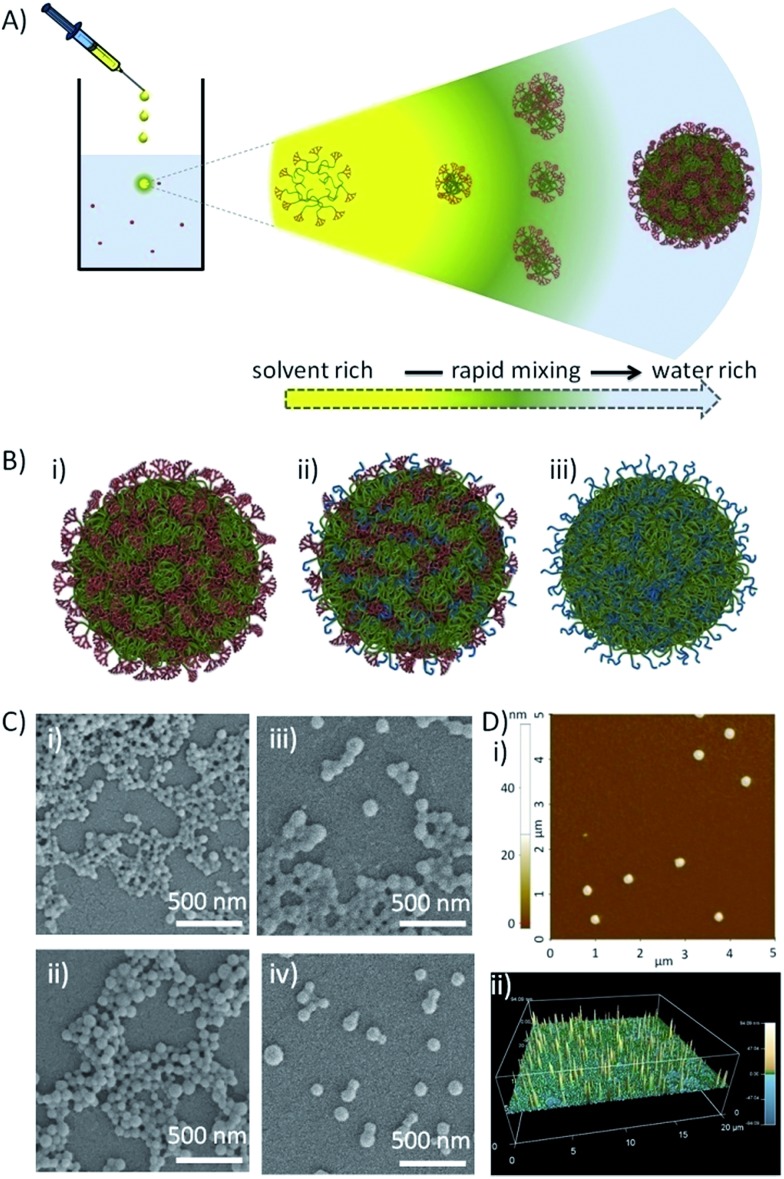
Nanoprecipitation of hyp-polydendrons. (A) Schematic of nano-precipitation from THF (good solvent) into water (poor solvent). Dissolved and expanded hyp-polydendrons collapse, forming self-assembling nuclei that form stable nanoparticles aqueous environments; (B) schematic representation of hyp-polydendron nanoprecipitates containing (i) 100% dendron initiator, (ii) a 50 : 50 molar ratio of G_2_ dendron–PEG_16_ initiators, and (iii) 100% PEG_16_ initiator; (C) scanning electron microscopy images of hyp-polydendron nanoprecipitates (initial concentration in THF = 5 mg mL^–1^, final aqueous concentration = 1 mg mL^–1^) with varying G_2_ dendron–PEG_16_ molar ratios: (i) 100 : 0, (ii) 90 : 10, (iii) 50 : 50, and (iv) 25 : 75; (D) atomic force microscopy of hyp-polydendron nanoprecipitate particles (initial concentration in THF = 5 mg mL^–1^, final aqueous concentration = 1 mg mL^–1^): (i) size image for nanoprecipitates generated from a G_2_ dendron–PEG_16_ molar ratio of 100 : 0, (ii) contour image of nanoprecipitates with a G_2_ dendron–PEG_16_ molar ratio of 25 : 75.

Subsequent organic solvent evaporation gave four aqueous nanoparticle dispersion concentrations (*C*_F_) for each material (2, 1, 0.1 and 0.05 mg mL^–1^), determined by the volume of the THF solution that was added to a fixed volume of water. Within each nanoprecipitation, the initial solvent droplet solution contains solvated branched polymer chains and, on addition to water, the THF-rich and water-rich phase boundary rapidly dilutes during diffusion of THF into the antisolvent (Fig. 2A). The branched vinyl polymer cores collapse to form nuclei that assemble to produce stabilized monodisperse nanoparticles within the aqueous phase.^38^ Characterisation was achieved using scanning electron (SEM) and atomic force microscopy (AFM; Fig. 2C and D) and dynamic light scattering (DLS; Fig. S6†). Unlike conventional nanoprecipitation, the assembled hyp-polydendron nanoparticles contain systematically varying peripheral dendrons and PEG_16_ chains at the nanoprecipitate surface (Fig. 2B(i)–(iii)), thereby opening considerable opportunities for future development.

The variation of nanoprecipitation conditions (polymer chemistry, polymer concentration and solvent : antisolvent dilution ratio) allowed control of the hyp-polydendron and hyp-polymer nanoprecipitate diameters (*z*-average diameters (*D*_*z*_) from 77–205 nm, Table 2). Zeta potentials (*ζ*) were also measured and showed negatively charged particles (Table 2); these values are consistent with earlier unfunctionalised nanoprecipitates.^49^ Narrow size distributions, with polydispersity indices (PdI) ranging from 0.058 to 0.140 (Table 2), are highly surprising given the very broad molecular weight distribution of the hyp-polydendron materials, Table 1. It may be assumed that significant architectural diversity is produced across the molecular weight distribution during synthesis, with variation of branch point placement leading to different branching densities and flexibilities of the internal core structures. Mixed initiator polymerization leads to considerable additional polymer complexity, including complication of the inherent distributions of molecular weight, architecture, branching density and G_2_–PEG_16_ ratios. Despite this high level of complexity, the near monodisperse nanoprecipitates showed remarkably uniform behavior and stability, with little detectable difference when stored in water for nearly a year (Table S1†). Addition of THF to the aqueous nanoprecipitates led to swelling of the nanoparticles without dissolution, even at high levels of THF (>37.5% v/v) (Fig. S7b†), whilst dilution of the nanoparticles with water led to no appreciable changes over considerable concentration ranges (Fig. S7a†).

Conversely, studies of nanoprecipitates derived from the analogous unbranched linear–dendritic hybrids that comprise the primary chain structure of the hyp-polydendrons, and PEG_16_-(HPMA)_50_ analogues (Table 1), led to broad size distributions after nanoprecipitation and increasing *D*_*z*_ and PdI (bimodal size distributions in some cases) over extended periods, with visual precipitation (Fig. S8†).

**Fig. 3:**
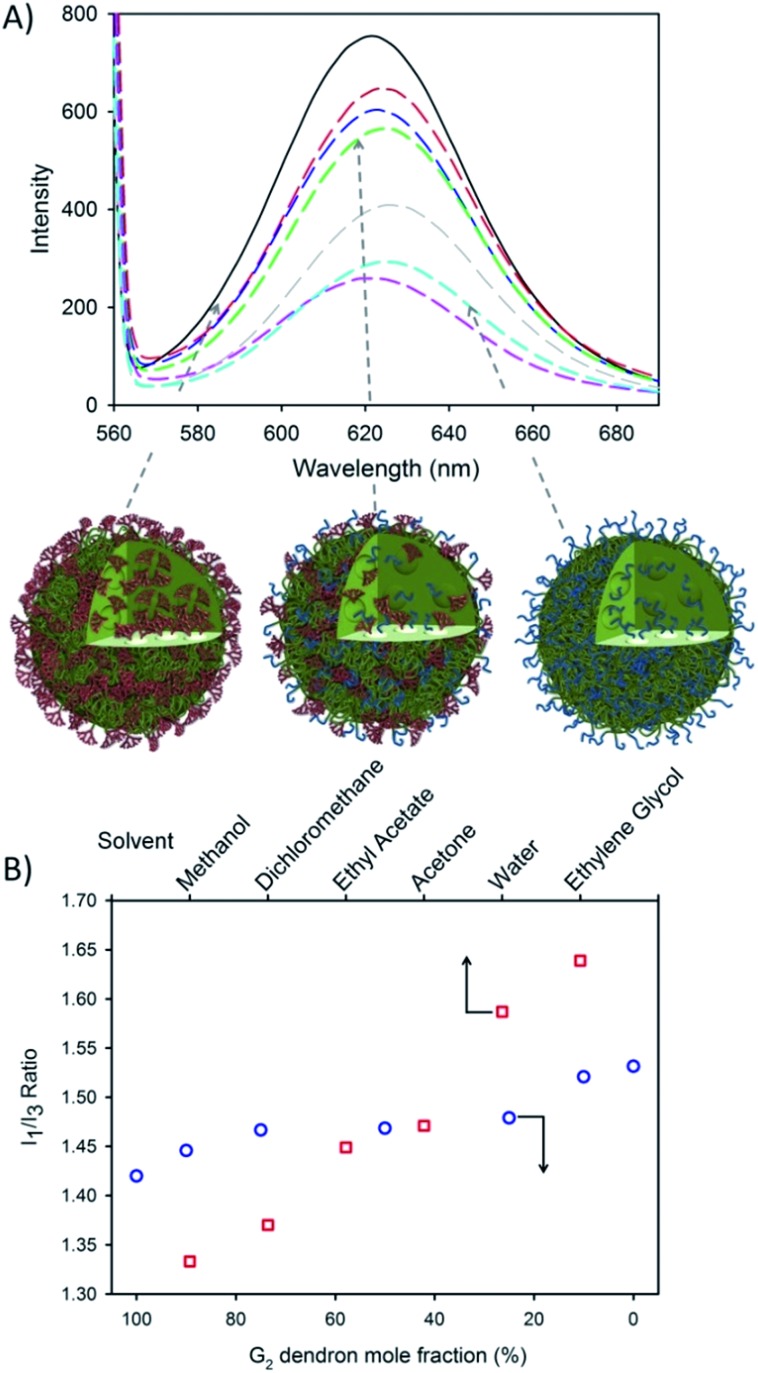
Encapsulation of hydrophobic guest molecules during nanoprecipitation of hyp-polydendrons with varying G_2_ dendron–PEG_16_ content. (A) Fluorimetric analysis of hyp-polydendron nanoprecipitates containing Nile Red produced from varying G_2_ dendron–PEG_16_ initiator ratios of 100 : 0 (solid black), 90 : 10 (red dash), 75 : 25 (blue dash), 50 : 50 (green dash), 27 : 75 (pink dash), 10 : 90 (grey dash) and 0 : 100 (cyan dash); (B) comparison of the observed *I*_1_/*I*_3_ ratio of pyrene within varying solvents (open red squares)^53,54^ and encapsulated within hyp-polydendrons synthesized using different G_2_ dendron–PEG_16_ initiator ratios (open blue circles).

Nile Red (NR) and pyrene encapsulation was accomplished by co-dissolving the fluorescent dyes into the branched polymer–THF solution of (0.1% w/w relative to polymer) prior to nanoprecipitation. These dyes were chosen due to their environment-dependent fluorescence and the ability to report on differences in lipophilicity (NR) or polarity (pyrene) within the core of the nanoprecipitates; no appreciable changes to *D*_*z*_ (Fig. S9 and S10†) were observed with encapsulated dye. NR emission (630 nm) was measured after excitation at 552 nm and was clearly dependent on the G_2_ dendron–PEG_16_ ratio (Fig. 3A).

This suggests the control of the environment within the hyp-polydendron nanoprecipitates whilst simultaneously modifying the expression of surface functionality. This is consistent with an assembly model involving aggregation of individual collapsed hyp-polydendron nuclei as complete entrapment of hyp-polydendrons within the nanoprecipitate would result (Fig. 3A), leading to internalization of varying internal ratios of G_2_ dendron and PEG_16_.

Further investigation of the internal nanoparticle environment was conducted *via* pyrene encapsulation. A similar variation of the internal nanoparticle environment was observed within the fine structure of pyrene fluorescence after excitation at 335 nm (Fig. S11†). The ratio of the intensities of the first and third vibrational bands (*I*_1_/*I*_3_) varied from 1.42–1.53, similar to pyrene fluorescence observed in solvents with polarities ranging from dichloromethane (*I*_1_/*I*_3_ = 1.37) to water (*I*_1_/*I*_3_ = 1.59) (Fig. 3B).^53,54^ The observed modification of the nanoparticle internal environment allows the potential for tuning of nanoparticle properties *via* initiator chemistry and/or monomer choice, providing considerable scope for tuning of physical properties. Similarly, modification of surface chemistry of the nanoprecipitate may also alter cellular or tissue interactions and this was investigated further with respect to model gut epithelium.

### Model epithelial permeation studies using aqueous hyp-polydendron nanoprecipitates

There are many clinically-utilized nanocarrier therapies benefiting patients globally, across diseases including cancer (*e.g.* Doxil and Myocet), fungal infection (*e.g.* Abelcet and AmBisome), hepatitis (*e.g.* Epaxal and Pegintron) and meningitis (*e.g.* DepoCyt).^55^ Dendrimers have been utilized as DNA transfection reagents (*e.g.* SuperFECT) and cancer/cardiac diagnostics (Stratus CS Acute Care Assay), whilst only limited dendrimer-derived therapeutics are being sought (*e.g.* VivaGel®).^55^

Nanocarriers are rarely dosed orally,^56^ but benefits may accrue in chronic diseases (*e.g.* HIV therapy or psychiatric disorders) where long-term daily dosing is required and repeated injection is not optimal for patients. The provision of an orally-administered nanocarrier, delivering nanoparticles to the circulation, may also provide cellular or tissue targeting benefits for acute diseases. Our aim within this study was to evaluate the potential for permeation through a gut epithelium model; therefore, a preliminary *in vitro* pharmacological evaluation of the behaviour of hyp-polydendron nanoprecipitates was conducted.

**Fig. 4:**
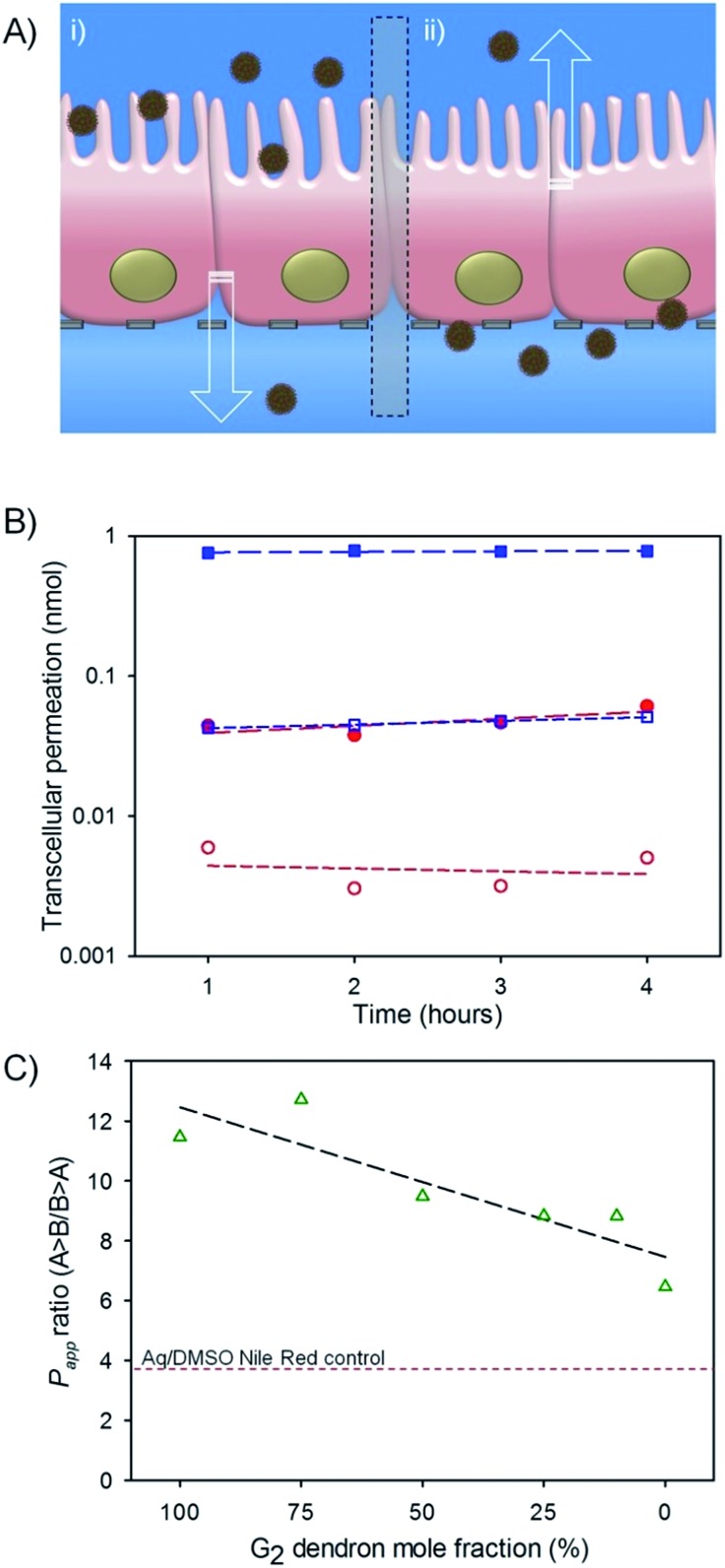
Preliminary evaluation of oral dosing potential for hyp-polydendron nanoprecipitates with varying G_2_ dendron–PEG_16_ content. (A) Schematic representation of hyp-polydendron nanoprecipitates passing from (i) apical (top) compartment (modelling the gut side) of a differentiated Caco-2 cell monolayer through to the basolateral (bottom) compartment (modelling the blood side) to evaluate potential for intestinal epithelium permeation, and (ii) movement of hyp-polydendron nanoprecipitates from the basolateral to the apical side of the monolayer; (B) comparison of permeation of a hyp-polydendron (10 : 90 G_2_ dendron–PEG_16_ ratio) nanoprecipitate (blue squares) and aqueous/DMSO solution (<1% v/v) of Nile Red (red circles) through a Caco-2 monolayer – B > A permeation (open symbols); A > B permeation (closed symbols) (C) apparent permeability (*P*_app_) of Nile Red when encapsulated in hyp-polydendron nanoprecipitates of varying G_2_ dendron–PEG_16_molar ratios (open green triangles) and as an aqueous/DMSO solution (<1% v/v) (red line).

Cytotoxicity of the seven aqueous nanoprecipitates was evaluated against the human epithelial colorectal adenocarcinoma (Caco-2) cell line (Fig. 4A) and no appreciable cytotoxicity was observed at achievable concentrations in assays assessing either adenosine triphosphate or 3-(4,5-dimethylthiazol-2-yl)-2,5-diphenyltetrazolium bromide (MTT) turnover (Fig. S12a–d†); as such the determination of an IC_50_ value for each polymer was not possible, indicating very low cytotoxicity towards Caco-2 cells. In the absence of obvious cytotoxicity to the gut model cell line, the transcellular permeation of hyp-polydendron nanoprecipitates (with encapsulated NR) was studied across differentiated Caco-2 monolayers as a model of absorption through the intestinal epithelium (Fig. 4A).^57^

Assessment of the apparent permeability (*P*_app_) of a substance is widely studied using this model, including the screening of discovery compounds within the pharmaceutical industry. *P*_app_ is a measurement of the permeation (flux) through the Caco-2 monolayer after normalization for the surface area of the membrane and the concentration of the study substance within the media.^58^ Although not fully representative of the *in vivo* material behaviour, this model uses a human cell line and provides indicative data to allow further development.

NR fluorescence monitoring was used to determine hyp-polydendron passage from the apical compartment of the transwell plate to the basolateral well (A > B; Fig. 4A(i)) which models gut to systemic circulation permeation. In addition, permeation studies from the basolateral to the apical compartment (B > A; Fig. 4A(ii)) was assessed. The *P*_app_ ratio is calculated as the ratio of (A > B)/(B > A), providing a relative indication of apparent oral absorption in the presence of active transport proteins (*e.g.* p-glycoprotein) known to limit oral bioavailability of many compounds. In all studies the confluence of the Caco-2 cell monolayer was assessed to ensure that an intact membrane was present throughout the experiments (see ESI†).

The A > B permeation of the hyp-polydendron nanoprecipitates with encapsulated NR was approximately an order of magnitude higher than observed B > A permeation in all cases (Table 3; Fig. 4B). Variable behavior of the various nanoprecipitates was also noted over the 4 hours study exposure with materials comprising 75%, 50% and 0% G_2_ dendron showing a steady increase in fluorescence within the receiver compartment, suggesting a continual permeation through the Caco-2 membrane (Fig. S13†). Surprisingly, *P*_app_ ratios across the materials were all considerably higher than the aqueous/DMSO solution of NR (Table 3) and were highly correlated with the molar content of dendron; decreasing linearly with decreasing dendron content (simple linear regression, *r* ^2^ = 0.78, *P* = 0.02; Fig. 4C). This data can also be interpreted as a possible impact of increasing PEG_16_ content leading to a decreasing overall permeation through the Caco-2 monolayers. However, the factors affecting the observed *P*_app_ ratio are clearly not straightforward as seen from the non-linear variation of either A > B or B > A permeation as the nanoprecipitate chemical composition varied, Table 3.

**Table 3:** Permeation of hyp-polydendron nanoprecipitates with varying G_2_–PEG_16_ molar ratios through Caco-2 monolayers

G_2_–PEG_16_ molar ratio	*P*_app_ (cm s^–1^)		*P*_app_ ratio (A > B)/(B > A)
	Apical > Basolateral (A > B)	Basolateral > Apical (B > A)	
100 : 0	1.763 × 10^–5^	1.538 × 10^–6^	11.4605
75 : 25	2.613 × 10^–5^	2.056 × 10^–6^	12.7123
50 : 50	5.271 × 10^–5^	5.555 × 10^–6^	9.4872
25 : 75	4.135 × 10^–5^	4.684 × 10^–6^	8.8279
10 : 90	4.042 × 10^–4^	4.580 × 10^–5^	8.8255
0 : 100	2.060 × 10^–5^	3.188 × 10^–6^	6.4626
Aq. Nile Red^*a*^	2.371 × 10^–5^	6.384 × 10^–6^	3.7140

^*a*^<1% v/v DMSO/water solution.

The mechanisms that underpin this correlation are not immediately obvious and further investigation is required to understand the relationship. The increased presence of short PEG_16_ chains appears to have a negative impact on the *P*_app_ ratio within this series of materials; however, the hyp-polymer (without G_2_ dendron) still provided a noticeable enhancement of permeation. These exploratory data indicate the potential for further tuning of behaviour and the production of orally dosed materials that deliver benefits as systemically circulating nanoparticles carrying encapsulated drugs.

## Conclusions

In summary, a strategy has been presented to synthesize a novel complex macromolecular architecture, hyperbranched polydendrons, with controlled surface functionality. Despite the non-uniform nature of the high molecular weight materials, monodisperse nanoprecipitates of tuneable size have been produced that are stable in water and encapsulate hydrophobic materials with control of internal nanoprecipitate environment. Exploratory studies have indicated the potential to mediate permeation through model human gut epithelium. This strategy offers synthetic ease and provides a route to the relatively simple formation of nanoparticles. Our ongoing research is focusing on manipulation of the structural components of the hyp-polydendron architecture to introduce additional chemical and physical functionality that is tuneable to specific pharmacological ambitions such as oral delivery and targeting.

## References

[cit1] Junghanns J.-U. A. H., Müller R. H. (2008). Int. J. Nanomed..

[cit2] Rabinow B. E. (2004). Nat. Rev. Drug Discovery.

[cit3] McDonald T. O., Giardiello M., Martin P., Siccardi M., Liptrott N. J., Smith D., Roberts P., Curley P., Schipani A., Khoo S. H., Long J., Foster A. J., Rannard S. P., Owen A. (2014). Adv. Healthcare Mater..

[cit4] Al-Jamal W. T., Kostarelos K. (2011). Acc. Chem. Res..

[cit5] Duncan R. (2011). Curr. Opin. Biotechnol..

[cit6] Ashley C. E., Carnes E. C., Phillips G. K., Padilla D., Durfee P. N., Brown P. A., Hanna T. N., Liu J., Phillilps B., Carter M. B., Carroll N. J., Jiang X., Dunphy D. R., Willman C. L., Petsev D. N., Evans D. G., Parikh A. N., Chackerian B., Wharton W., Peabody D. S., Brinker C. J. (2011). Nat. Mater..

[cit7] Stuurman F. E., Nuijen B., Beijnen J. H., Schellens J. H. M. (2013). Clin. Pharmacokinet..

[cit8] Siddiqui M. D. N., Garg G., Sharma P. K. (2011). Adv. Biol. Res..

[cit9] Boffito M., Jackson A., Owen A., Becker S. (2014). Drugs.

[cit10] van't Klooster G., Hoeben E., Borghys H., Looszova A., Bouche M.-P., van Velsen F., Baert L. (2010). Antimicrob. Agents Chemother..

[cit11] Spreen W., Min S., Ford S. L., Chen S., Lou Y., Bomar M., St Clair M., Piscitelli S., Fujiwara T. (2013). HIV Clin. Trials.

[cit12] Branco M. C., Schneider J. P. (2009). Acta Biomater..

[cit13] Gaucher G., Satturwar P., Jones M.-C., Furtos A., Leroux J.-C. (2010). Eur. J. Pharm. Biopharm..

[cit14] Bromberg L. (2008). J. Controlled Release.

[cit15] Pepić I., Lovrić J., Filipović-Grčić J. (2013). Eur. J. Pharm. Sci..

[cit16] Mei L., Zhang Z., Zhao L., Huang L., Yang X.-L., Tang J., Feng S.-S. (2013). Adv. Drug Delivery Rev..

[cit17] Grayson S. M., Fréchet J. M. J. (2001). Chem. Rev..

[cit18] Röglin L., Lempens E. H. M., Meijer E. W. (2011). Angew. Chem., Int. Ed..

[cit19] Lee C. C., MacKay J. A., Fréchet J. M. J., Szoka F. (2005). Nat. Biotechnol..

[cit20] Wurm F., Frey H. (2011). Prog. Polym. Sci..

[cit21] Hermans T. M., Broeren M. A. C., Gomopoulos N., van der Schoot P., van Genderen M. H. P., Sommerdijk N. A. J. M., Fytas G., Meijer E. W. (2009). Nat. Nanotechnol..

[cit22] Rannard S. P., Davis N. J. (2000). J. Am. Chem. Soc..

[cit23] Rannard S., Davis N., McFarland H. (2000). Polym. Int..

[cit24] Aulenta F., Drew M. G. B., Foster A., Hayes W., Rannard S., Thornthwaite D. W., Worrall D. R., Youngs T. G. A. (2005). J. Org. Chem..

[cit25] Diallo M. S., Balogh L., Shafagati A., Johnson J. H., Goddard W. A., Tomalia D. A. (1999). Environ. Sci. Technol..

[cit26] Carlmark A., Malmström E., Malkoch M. (2013). Chem. Soc. Rev..

[cit27] Stoddart A., Feast W. J., Rannard S. P. (2012). Soft Matter.

[cit28] Ortega P., Serramia M. J., Munoz-Fernandez M. A., de la Mata F. J., Gomez R. (2010). Tetrahedron.

[cit29] Astruc D., Boisselier E., Ornelas C. (2010). Chem. Rev..

[cit30] Oliveira J. M., Salgado A. J., Sousa N., Mano J. F., Reis R. L. (2010). Prog. Polym. Sci..

[cit31] Menjoge A. R., Kannan R. M., Tomalia D. A. (2010). Drug Discovery Today.

[cit32] Lim J., Kostiainen M., Maly J., da Costa V. C. P., Annunziata O., Pavan G. M., Simanek E. E. (2013). J. Am. Chem. Soc..

[cit33] Chang Y., Kim C. (2001). J. Polym. Sci., Part A-1: Polym. Chem..

[cit34] O'Brien N., McKee A., Sherrington D. C., Slark A. T., Titterton A. (2000). Polymer.

[cit35] Weaver J. V. M., Rannard S. P., Cooper A. I. (2009). Angew. Chem., Int. Ed..

[cit36] He T., Adams D. J., Butler M. F., Yeoh C. T., Cooper A. I., Rannard S. P. (2007). Angew. Chem., Int. Ed..

[cit37] He T., Adams D. J., Butler M. F., Cooper A. I., Rannard S. P. (2009). J. Am. Chem. Soc..

[cit38] Hatton F. L., Chambon P., McDonald T. O., Owen A., Rannard S. P. (2014). Chem. Sci..

[cit39] Kolhatkar R. B., Kitchens K. M., Swaan P. W., Ghandehari H. (2007). Bioconjugate Chem..

[cit40] Willcock H., Cooper A. I., Adams D. J., Rannard S. P. (2009). Chem. Commun..

[cit41] Huang B., Kukowska-Latallo J. F., Tang S., Zong H., Johnson K. B., Desai A., Gordon C. L., Leroueil P. R., Baker J. R. (2012). Bioorg. Med. Chem. Lett..

[cit42] Hawker C. J., Fréchet J. M. J. (1992). J. Am. Chem. Soc..

[cit43] Yoon K., Goyal P., Weck M. (2007). Org. Lett..

[cit44] Antoni P., Hed Y., Nordberg A., Nystrom D., von Holst H., Hult A., Malkoch M. (2009). Angew. Chem., Int. Ed..

[cit45] Thioune O., Fessi H., Devissaguet J. P., Puisieux F. (1997). Int. J. Pharm..

[cit46] Schubert S., Delaney J. T., Schubert U. S. (2011). Soft Matter.

[cit47] Lamer V. K., Dinegar R. H. (1950). J. Am. Chem. Soc..

[cit48] Zhang C., Pansare V. J., Prud'homme R. K., Priestly R. D. (2012). Soft Matter.

[cit49] Slater R. A., McDonald T. O., Adams D. J., Draper E. R., Weaver J. V. M., Rannard S. P. (2012). Soft Matter.

[cit50] Valencia P. M., Basto P. A., Zhang L., Rhee M., Langer R., Farokhzad O. C., Karnik R. (2010). ACS Nano.

[cit51] Cheng J., Teply B. A., Sherifi I., Sung J., Luther G., Gu F. X., Levy-Nissenbaum E., Radovic-Moreno A. F., Langer R., Farokhzad O. C. (2007). Biomaterials.

[cit52] Shi J., Xiao Z., Kamaly N., Farokhzad O. C. (2011). Acc. Chem. Res..

[cit53] Dong D. C., Winnik M. A. (1982). Photochem. Photobiol..

[cit54] Kalyanasundaram K., Thomas J. K. (1977). J. Am. Chem. Soc..

[cit55] Duncan R., Gaspar R. (2011). Mol. Pharm..

[cit56] Maher S., Leonard T. W., Jacobsen J., Brayden D. J. (2009). Adv. Drug Delivery Rev..

[cit57] Profit L., Eagling V. A., Back D. J. (1999). AIDS.

[cit58] Palumbo P., Picchini U., Beck B., van Gelder J., Delbar N., DeGaetano A. (2008). J. Pharmacokinet. Pharmacodyn..

