# Supplementary material for: Hyperbranched polydendrons: a new nanomaterials platform with tuneable permeation through model gut epithelium
Source: Chem Sci. 2014 Oct 3;6(1):326–34. doi: 10.1039/c4sc02889a (PMC5586204; doi:10.1039/c4sc02889a)
Supplement: Supplementary file 1 [file SC-006-C4SC02889A-s001.pdf]

Electronic Supplementary Material (ESI) for Chemical Science.
This journal is © The Royal Society of Chemistry 2014

Hyperbranched Polydendrons: a new nanomaterials platform with tuneable permeation through model gut epithelium

Fiona L. Hatton,[†] Lee M. Tatham,[ɸ] Louise R. Tidbury,[ɸ] Pierre Chambon,[†] Tao He,[‡] Andrew Owen[ɸ,*] and Steven P. Rannard.[†,*]

[†]Department of Chemistry, University of Liverpool, Crown Street, L69 3BX, UK

[ɸ]Department of Molecular and Clinical Pharmacology, University of Liverpool, Block H, 70 Pembroke Place, Liverpool, L69 3GF, UK

[‡]Institute of Chemical and Engineering Sciences, Agency for Science, Technology and Research (A*STAR), 1, Pesek Road, Jurong Island, Singapore, 627833

Supplementary Information

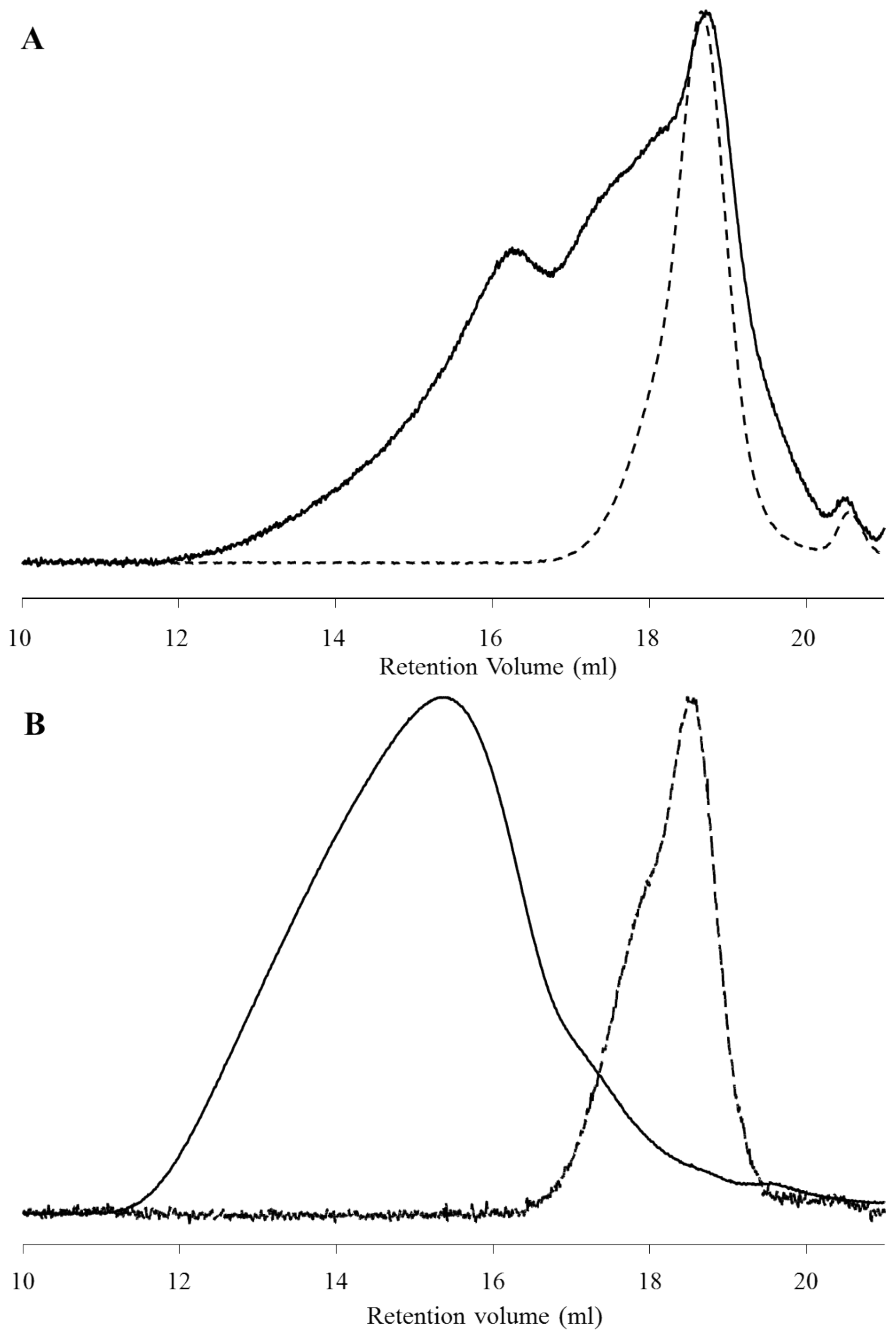

Figure S1 – Triple detection Gel Permeation Chromatography (GPC) overlays of A) refractive index detector and B) right angle light scattering detector for **G_2** Dendron initiated ATRP of HPMA – target DP_n = 50 monomer units. The figures show analysis of both linear dendritic polymer hybrids (dotted lines) and branched polymerisations (solid line). A small shoulder is seen in the RALS signal for the linear-dendritic polymer analogue, possibly due to a small amount of bifunctional monomer within the commercial sample as purchased.

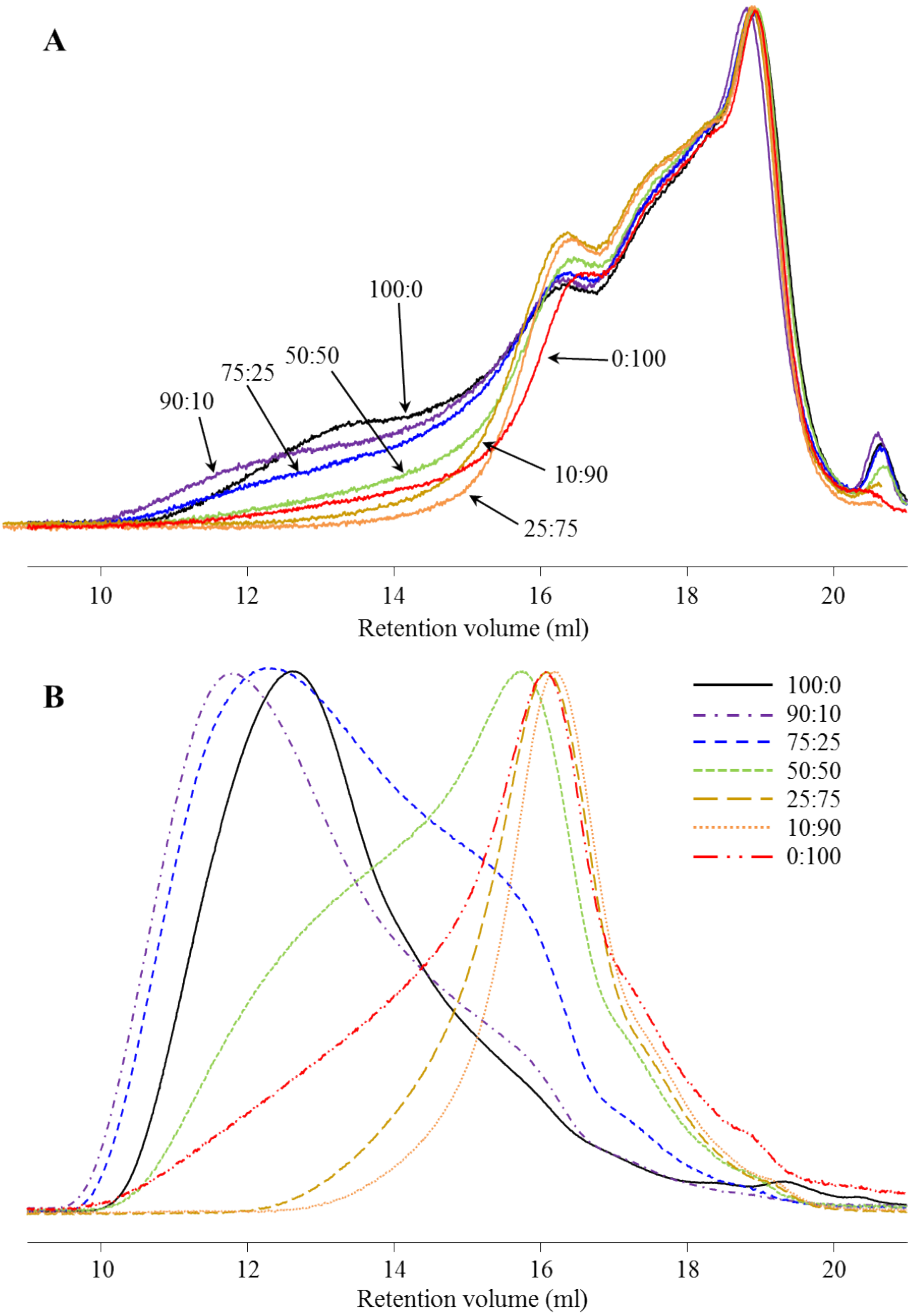

Figure S2 - Gel permeation chromatography (GPC) overlays of A) refractive index detector and B) right angle light scattering detector for the Polydendron series with varying initiator ratios

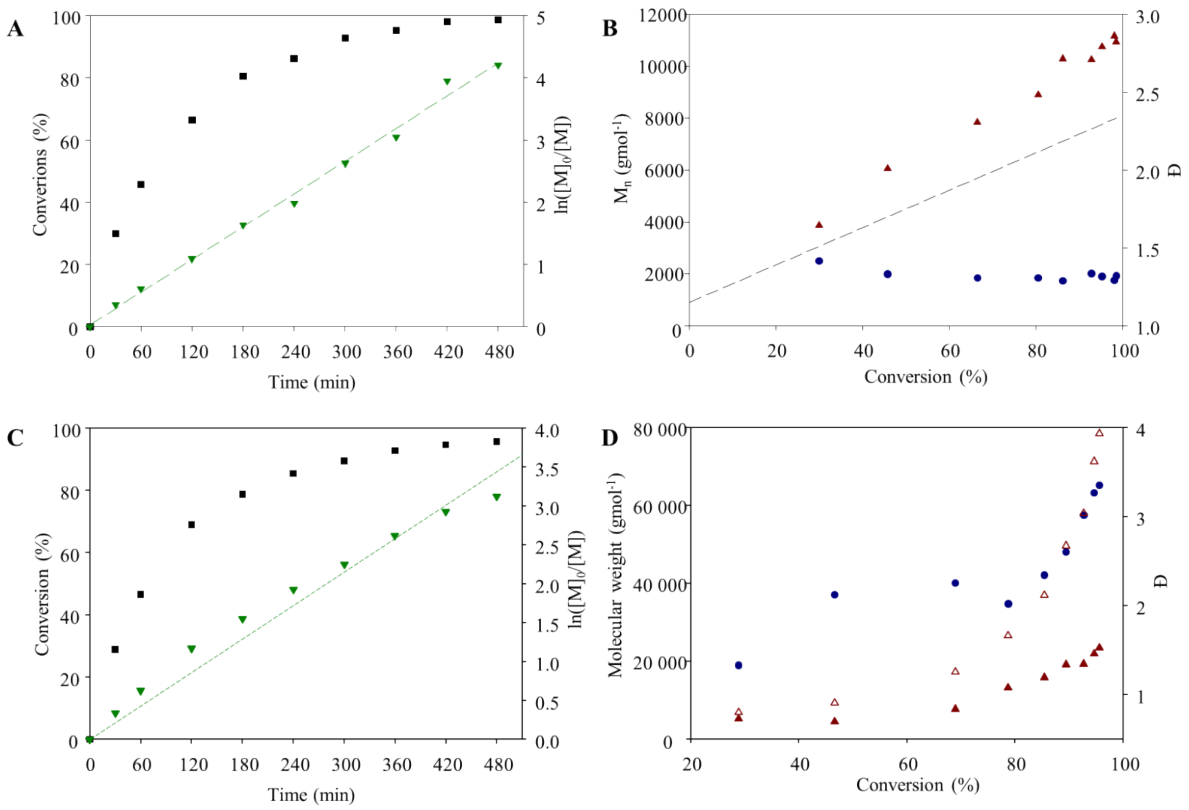

Figure S3 - Kinetic experiment data for the PEG_{16}-Br initiated polymerisations. PEG_{16}-$pHPMA_{50}$: A and B, PEG_{16}-$pHPMA_{50}$-$EGDMA_{0.8}$: C and D. Conversion (black squares) and $\ln([M]_0/[M])$ (green down triangles), M_n (red up triangles), M_w (red empty up triangles) theoretical Mn (black dotted line) and Đ (blue circles)

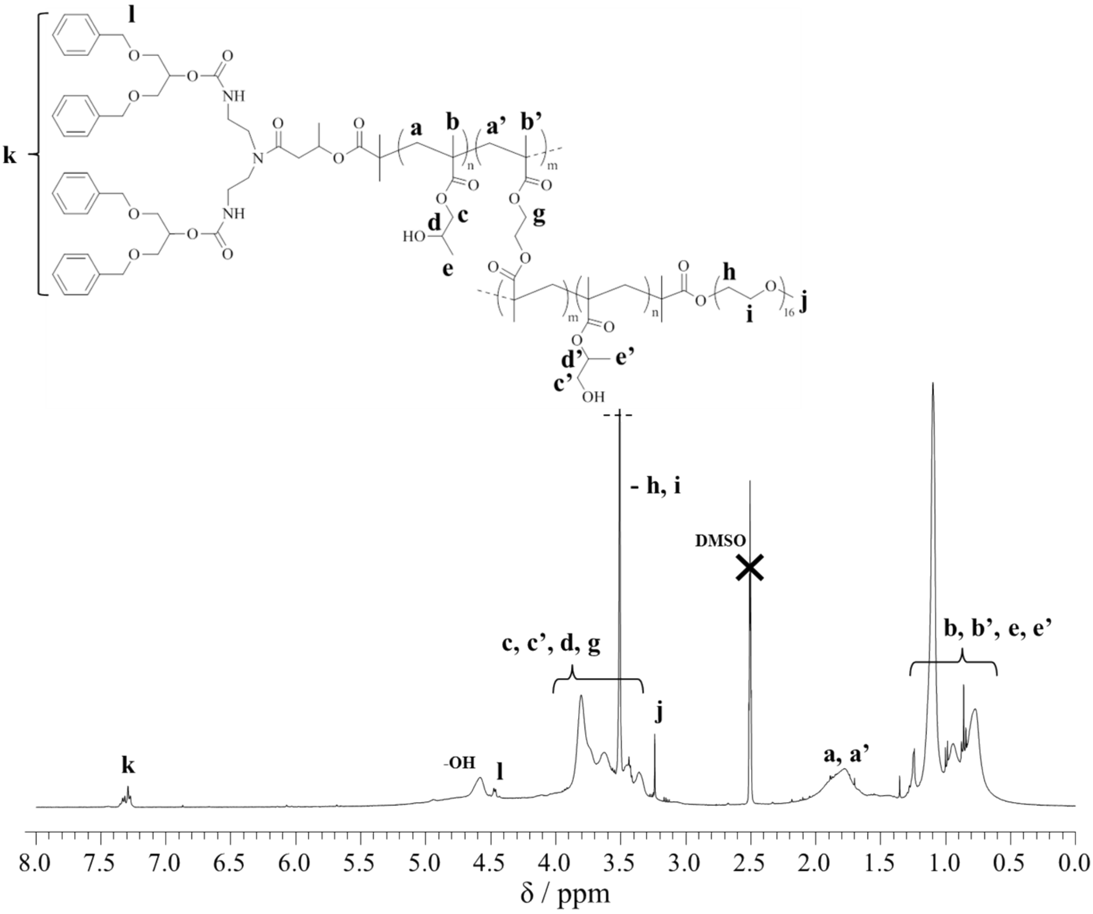

Figure S4a - ^{1}H NMR spectrum (400 MHz, d_6-DMSO) of 25:75-pHPMA$_{50}$-EGDMA$_{0.8}$ with major peaks assigned

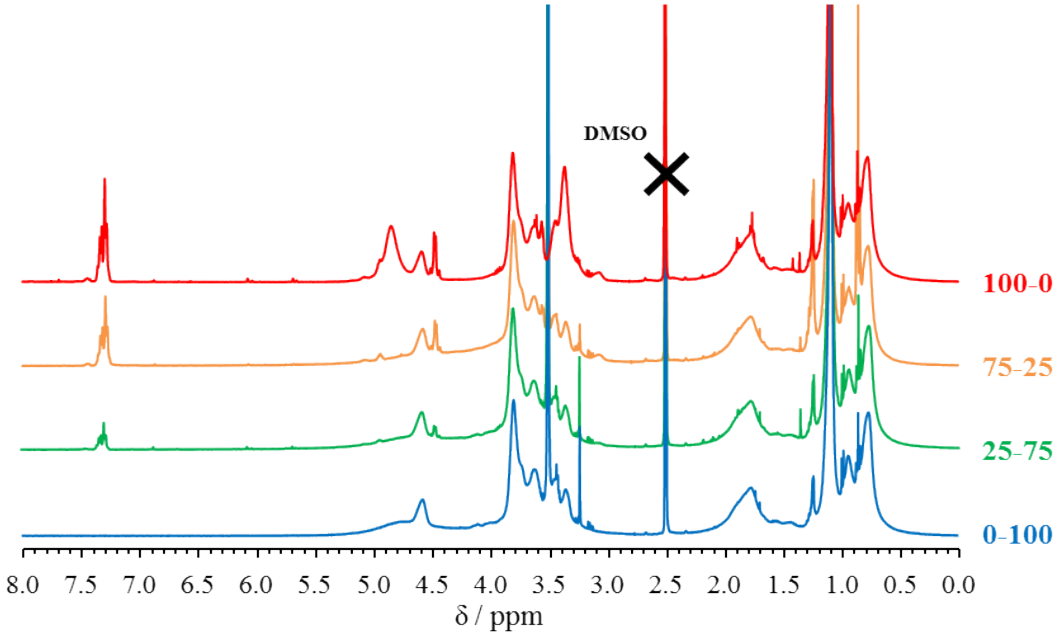

Figure S4b -^{1}H NMR (400 MHz, d_6-DMSO) spectra overlay for 100:0, 75:25, 25:75 and 0:100 –pHPMA$_{50}$-EGDMA$_{0.8}$ polymers

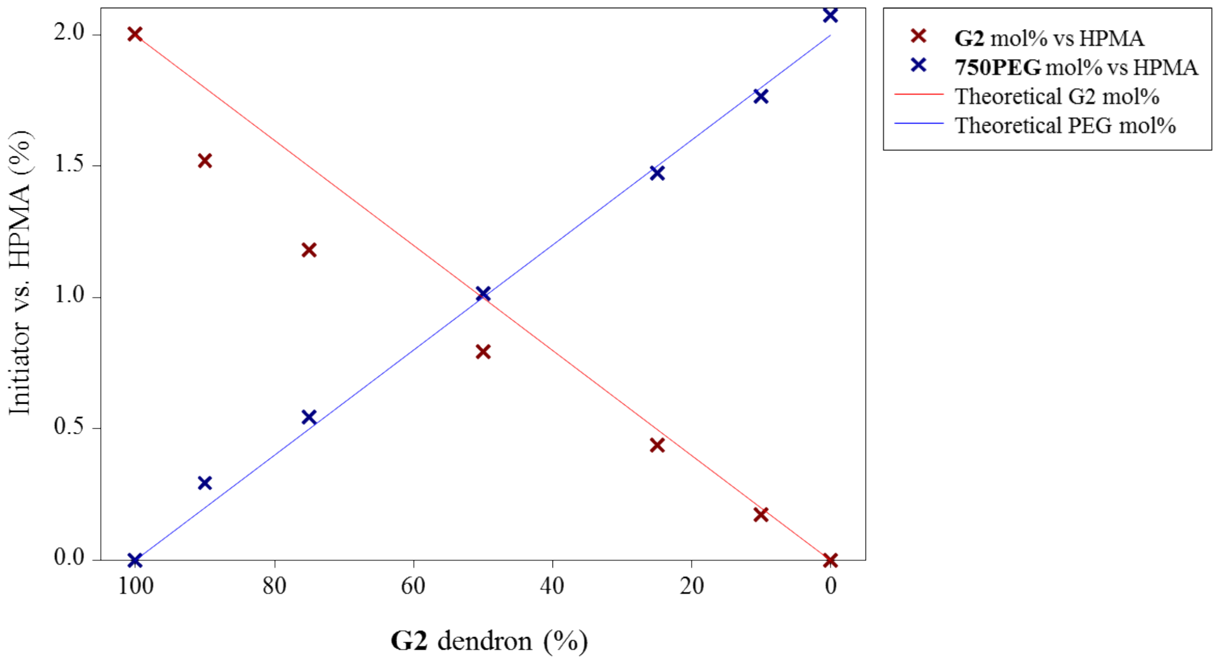

Figure S5 - Initiator mol% *vs*. HPMA mol% for each *hyp*-polydendron observed by ^{1}H NMR spectroscopy (blue and red crosses) and the theoretical values (blue and red lines)

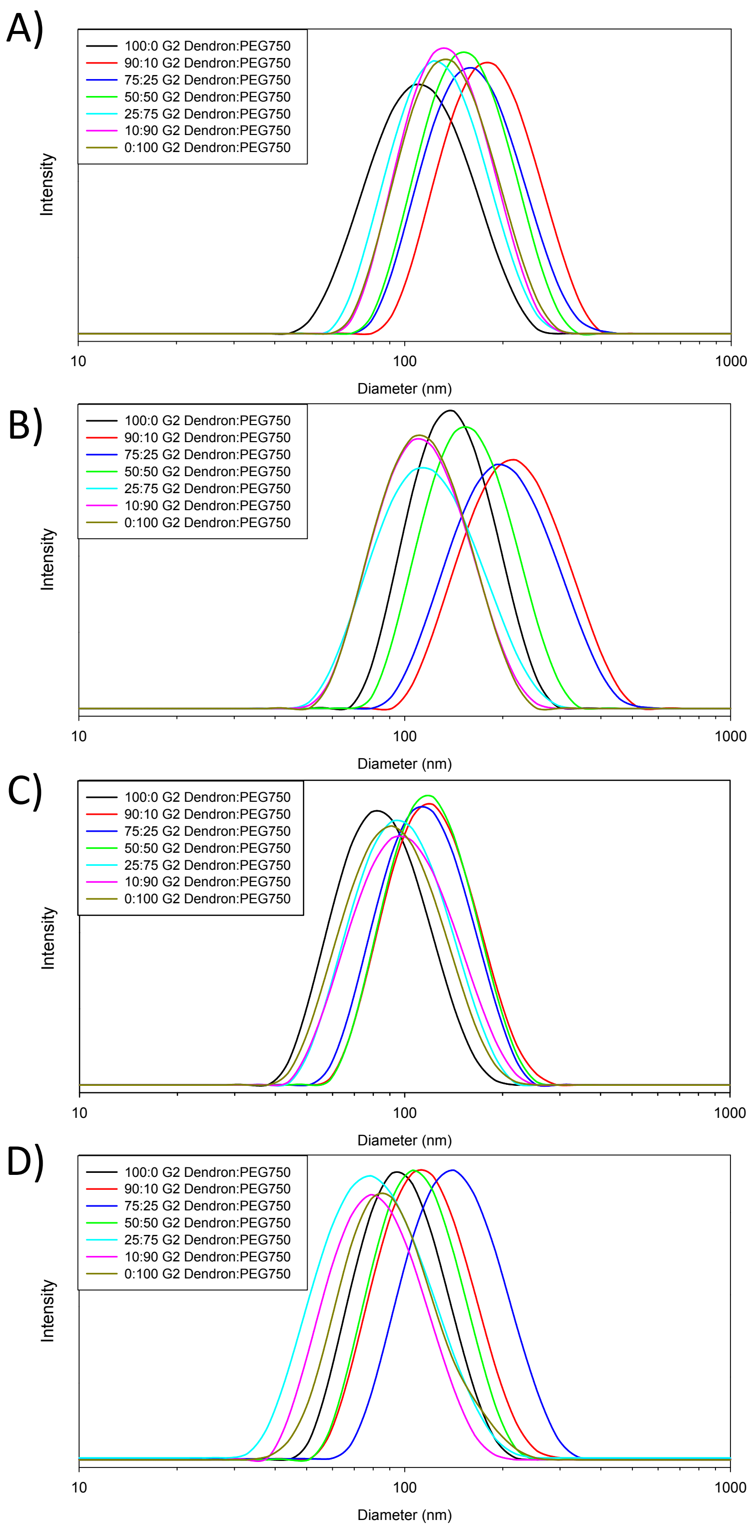

Figure S6 – Dynamic Light Scattering (DLS) of nanoprecipitated polydendrons containing varying $\mathbf{G_2}$ Dendron:PEG$_{16}$ratios. A) Initial THF concentration = 10 mg/mL, final aqueous concentration = 2 mg/mL; B) Initial THF concentration = 10 mg/mL, final aqueous concentration = 0.1 mg/mL; C) Initial THF concentration = 5 mg/mL, final aqueous concentration = 1 mg/mL; D) Initial THF concentration = 5 mg/mL, final aqueous concentration = 0.05 mg/mL

Table S1 -D_z and PdI measured by DLS for **G$_2$**:PEG$_{16}$-pHPMA$_{50}$-EGDMA$_{0.8}$ samples produced from an initial concentration of 5 mg/mL and a final concentration of 1 mg/mL (***i$_5$-f$_1$***), stored for over 11 months

**G$_2$**:PEG$_{16}$-pHPMA$_{50}$-EGDMA$_{0.8}$	***i$_5$-f$_1$***		***i$_5$-f$_1$ + 11 months***	
	D_z (nm)	PdI	D_z (nm)	PdI
100:0	58	0.096	60	0.108
90:10	90	0.030	89	0.030
75:25	71	0.082	69	0.037
50:50	68	0.056	68	0.050
25:75	82	0.060	91	0.190
10:90	63	0.103	68	0.118
0:100	140	0.058	145	0.051

D_z = z-average diameter

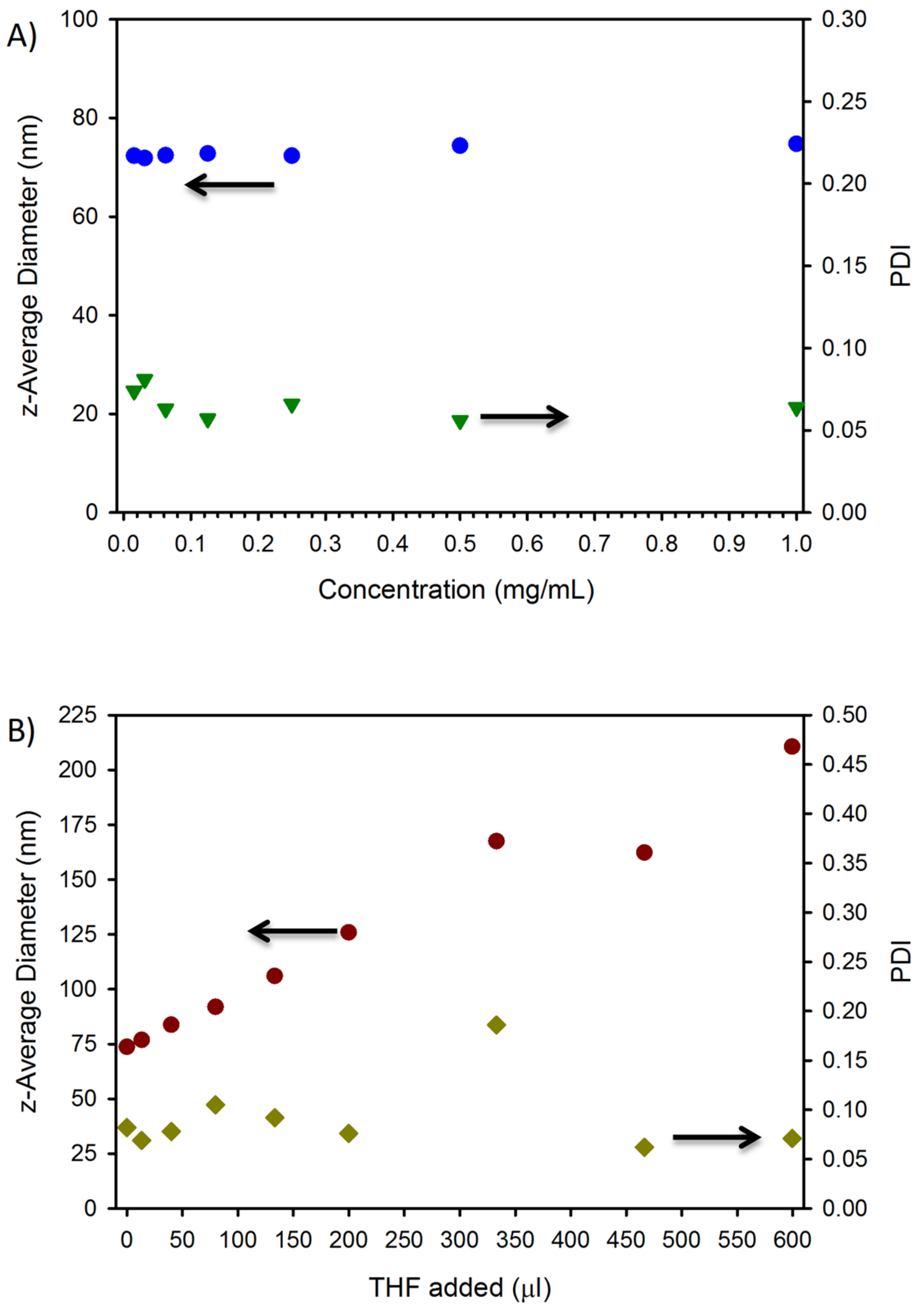

Figure S7 – Stability of *hyp*-polydendron nanoprecipitates, derived from polymers containing a $\mathbf{G_2}$ Dendron:PEG_{16} molar ratio of 50:50, to A) dilution with water, and B) addition of a good solvent (THF).

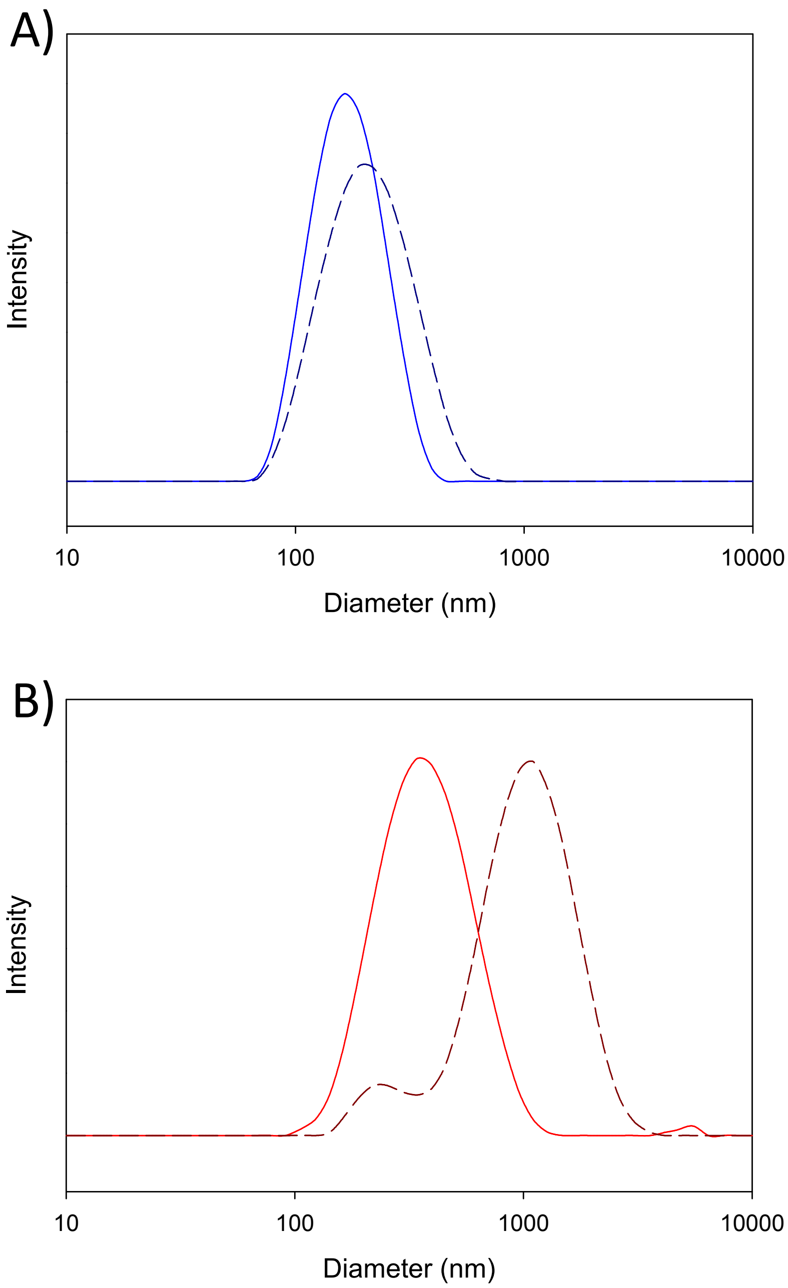

Figure S8 – Dynamic Light Scattering study of nanoprecipitate stability. Nanoprecipitation of linear HPMA polymers (target DP_n = 50 monomer units) initiated with A)**G_2**dendron**1**, and B) PEG_{16}initiators. Nanoprecipitates were prepared from an initial THF concentration = 5 mg/mL, resulting in a final aqueous concentration = 1 mg/mL. Solid lines indicate nanoprecipitates measured after solvent evaporation and dashed lines indicate the same aqueous nanoprecipitates after 30 days at ambient temperature.

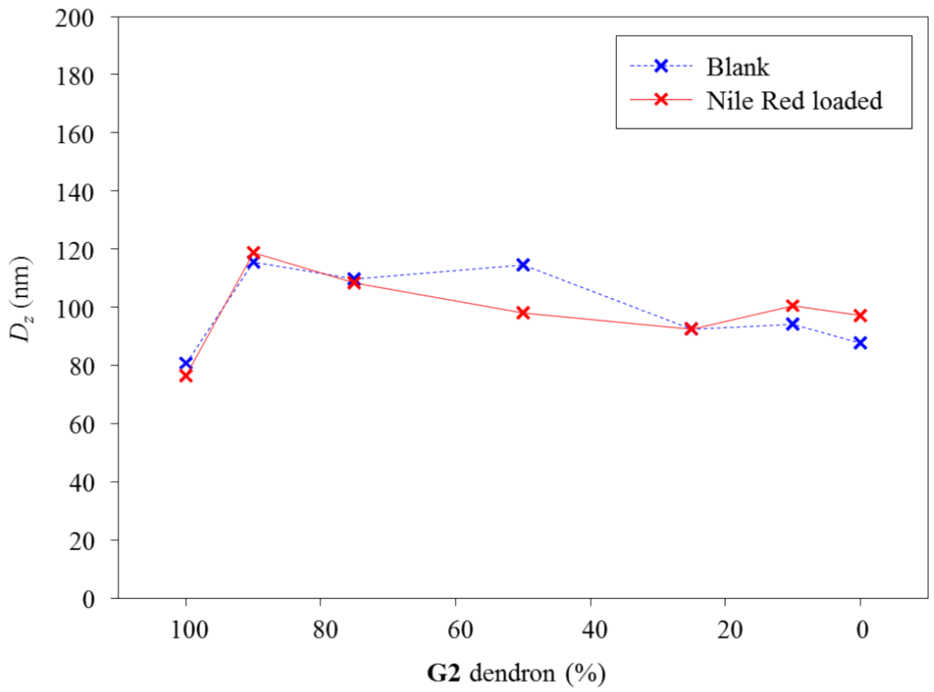

Figure S9a- Nanoparticle sizes for **G_2**:PEG_{16}-$pHPMA_{50}$-$EGDMA_{0.8}$ blank nanoparticles (blue crosses) and with 0.1 w/w% Nile red encapsulated (red crosses) formulated with an initial concentration of 5 mg/mL and final concentration of 1 mg/mL (i_5-f_1).

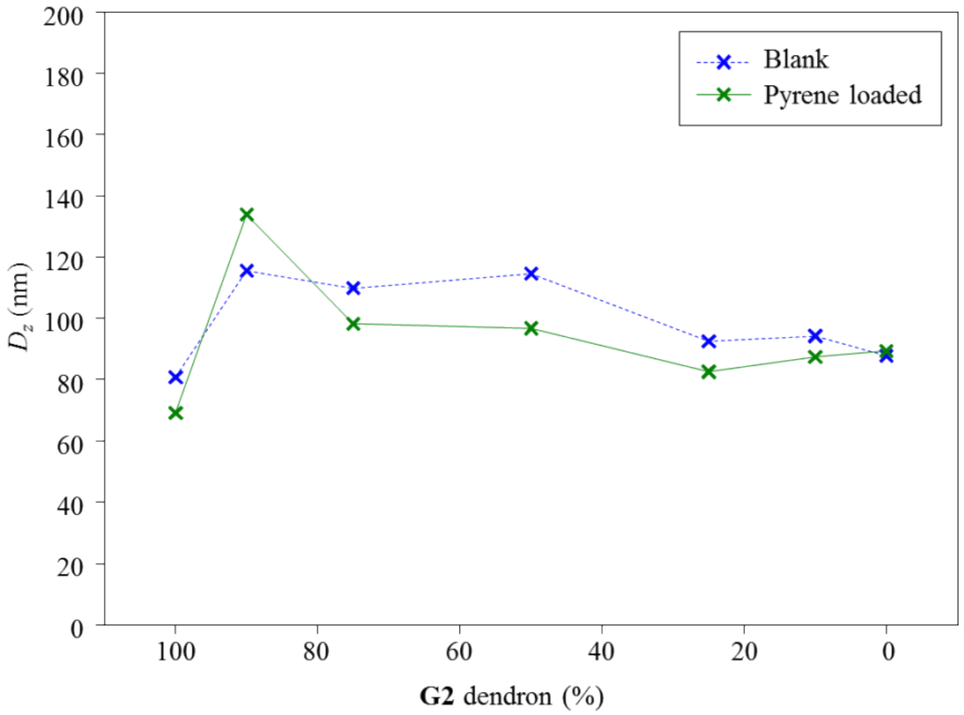

Figure S9b- Nanoparticle sizes for **G_2**:PEG_{16}-$pHPMA_{50}$-$EGDMA_{0.8}$ blank nanoparticles (blue crosses) and with 0.1 w/w% pyrene encapsulated (red crosses) formulated with an initial concentration of 5 mg/mL and final concentration of 1 mg/mL (i_5-f_1).

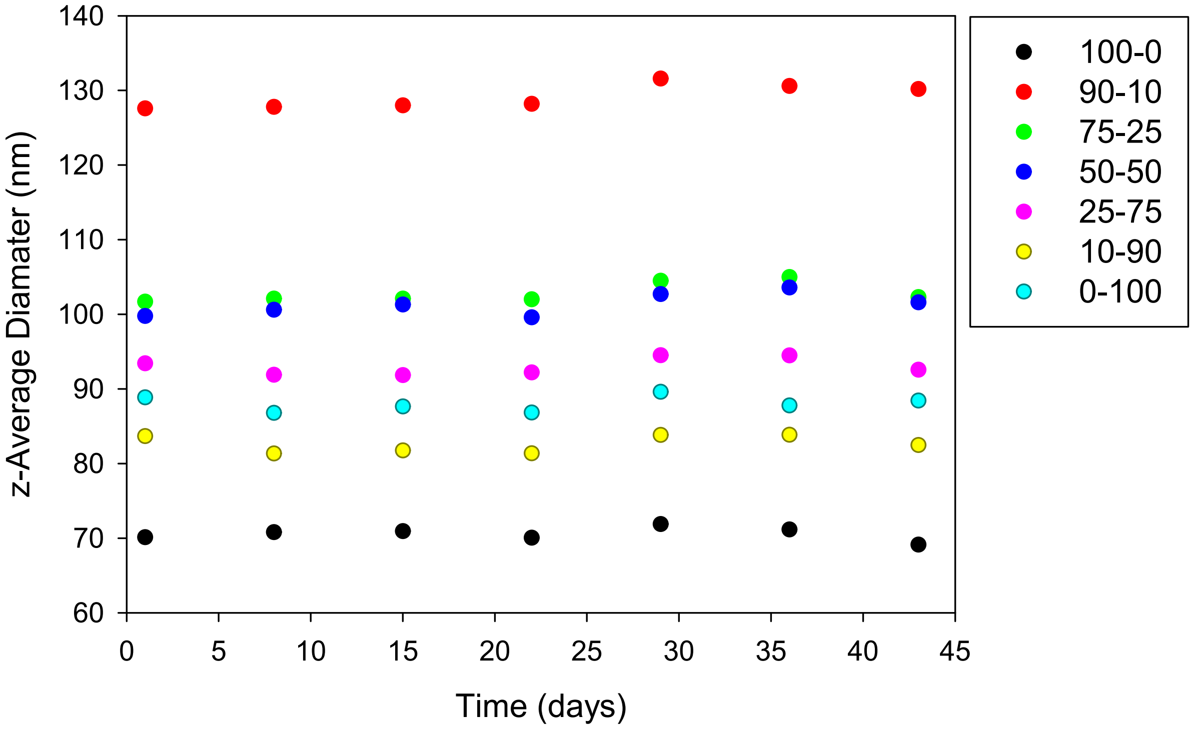

Figure S10 – Dynamic Light Scattering study of nanoprecipitate stability in water at ambient temperature. Nanoprecipitation of branched HPMA polymers (target DP_n = 50 monomer units) with varying $\mathbf{G_2}$ dendron:PEG_{16} molar ratios (shown in legend) and prepared from an initial THF concentration = 5 mg/mL and resulting in a final aqueous concentration = 1 mg/mL.

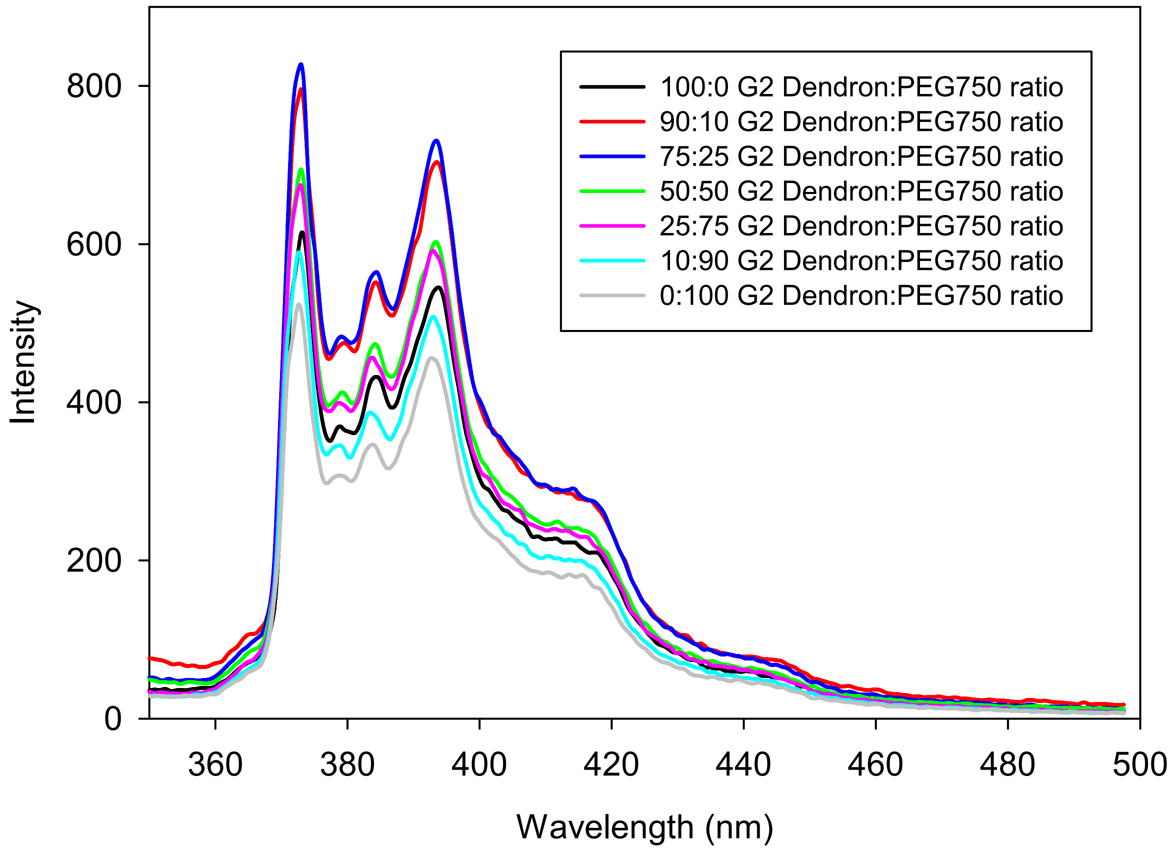

Figure S11– Fluorimetric analysis of pyrene containing branched polymer nanoprecipitates with varying ratios of $\mathbf{G_2}$ dendron (**1**) and PEG_{16}(**4**) initiators. Nanoprecipitates were prepared from an initial THF concentration = 5 mg/mL, resulting in a final aqueous concentration = 1 mg/mL.

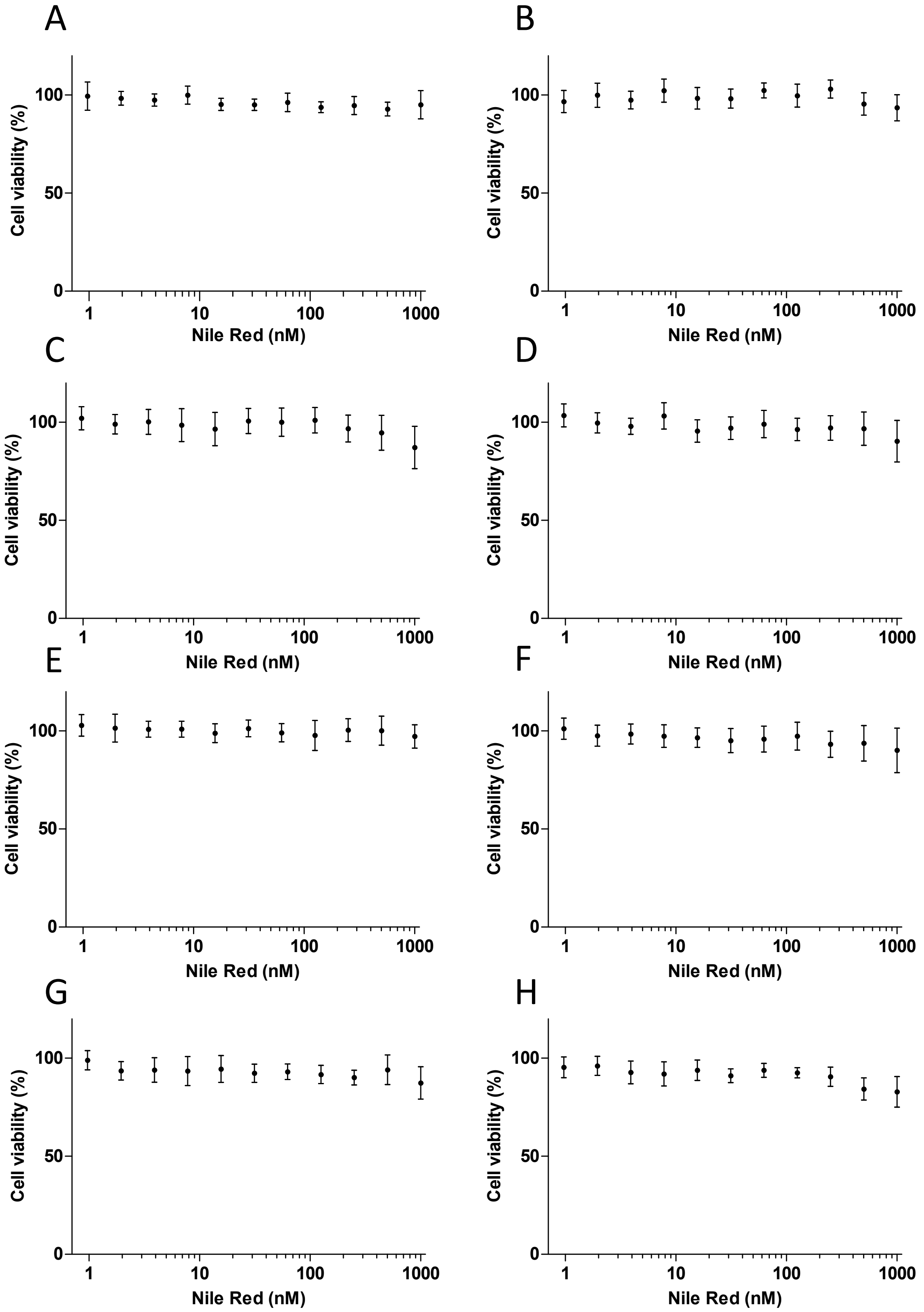

Figure S12a - MTT assay of Caco-2 cells following 24 hour incubation with aqueous Nile Red and each polydendron. A = aqueous Nile Red, EC_{50} 1.160. B = **G_2**Dendron:PEG_{16} 0:100, EC_{50} 2.509. C = **G_2** Dendron:PEG_{16}10:90, EC50 1.410. D = **G_2** Dendron:PEG_{16}25:75, EC_{50} 1.567. E = **G_2** Dendron:PEG_{16}50:50, EC_{50} 1.083. F = **G_2** Dendron:PEG_{16}75:25, EC_{50} 1.565, G = **G_2** Dendron:PEG_{16} 90:10, EC_{50} 1.607. H = **G_2** Dendron:PEG_{16}100:0, EC_{50} 2.678.

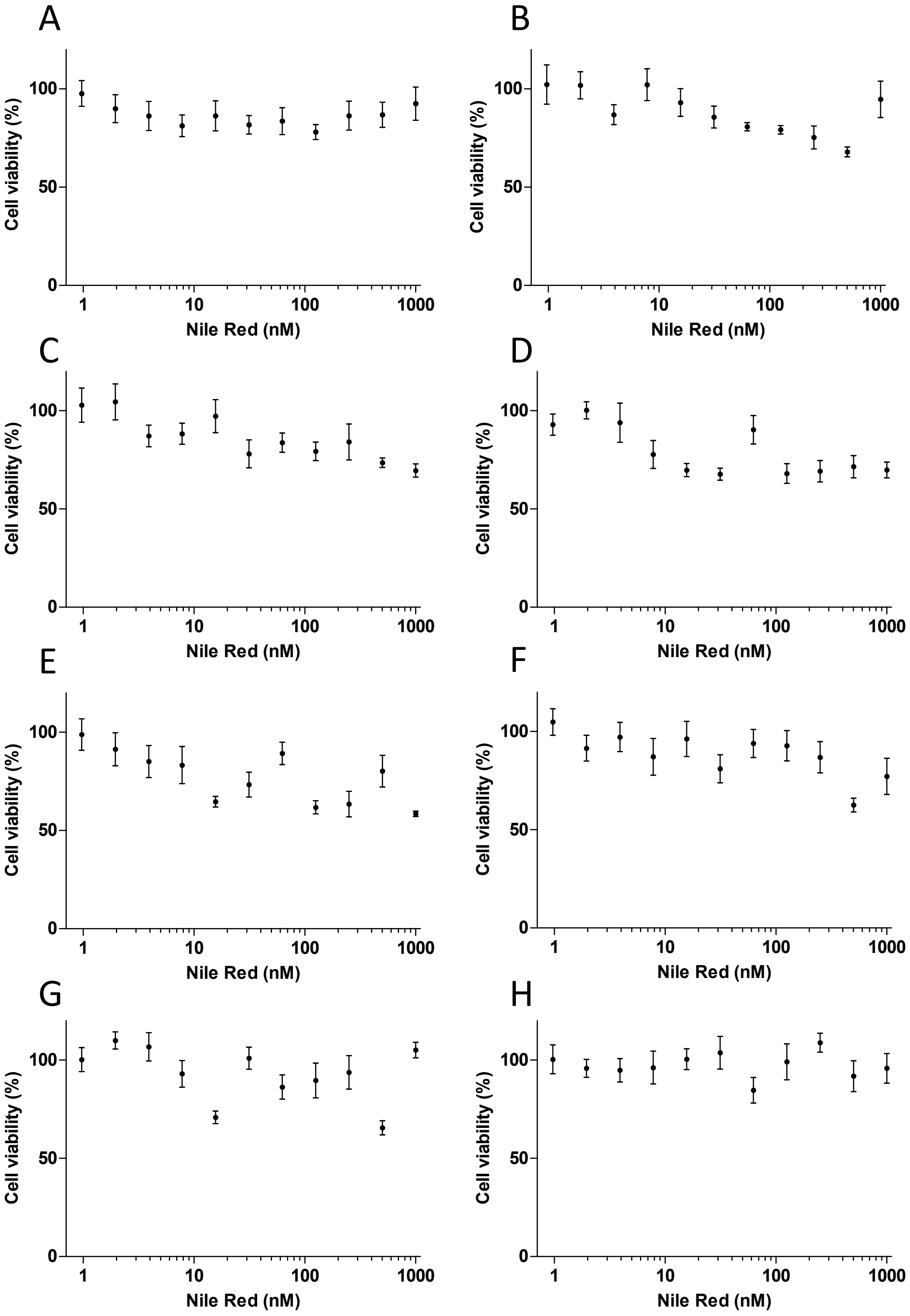

Figure S12b - MTT assay of Caco-2 cells following 120 hour incubation with aqueous Nile Red and each polydendron. A = aqueous Nile Red, No EC_{50}. B = $\mathbf{G_2}$ Dendron:PEG_{16}0:100, EC_{50} 1.528. C = $\mathbf{G_2}$ Dendron:PEG_{16}10:90, No EC_{50}. D = $\mathbf{G_2}$ Dendron:PEG_{16}25:75, EC_{50} 6.166. E = $\mathbf{G_2}$ Dendron:PEG_{16}50:50, EC_{50} 0.7856. F =$\mathbf{G_2}$ Dendron:PEG_{16} 75:25, No EC_{50}, G = $\mathbf{G_2}$ Dendron:PEG_{16}90:10, EC_{50} 0.2176. H = $\mathbf{G_2}$ Dendron:PEG_{16}100:0, No EC_{50}.

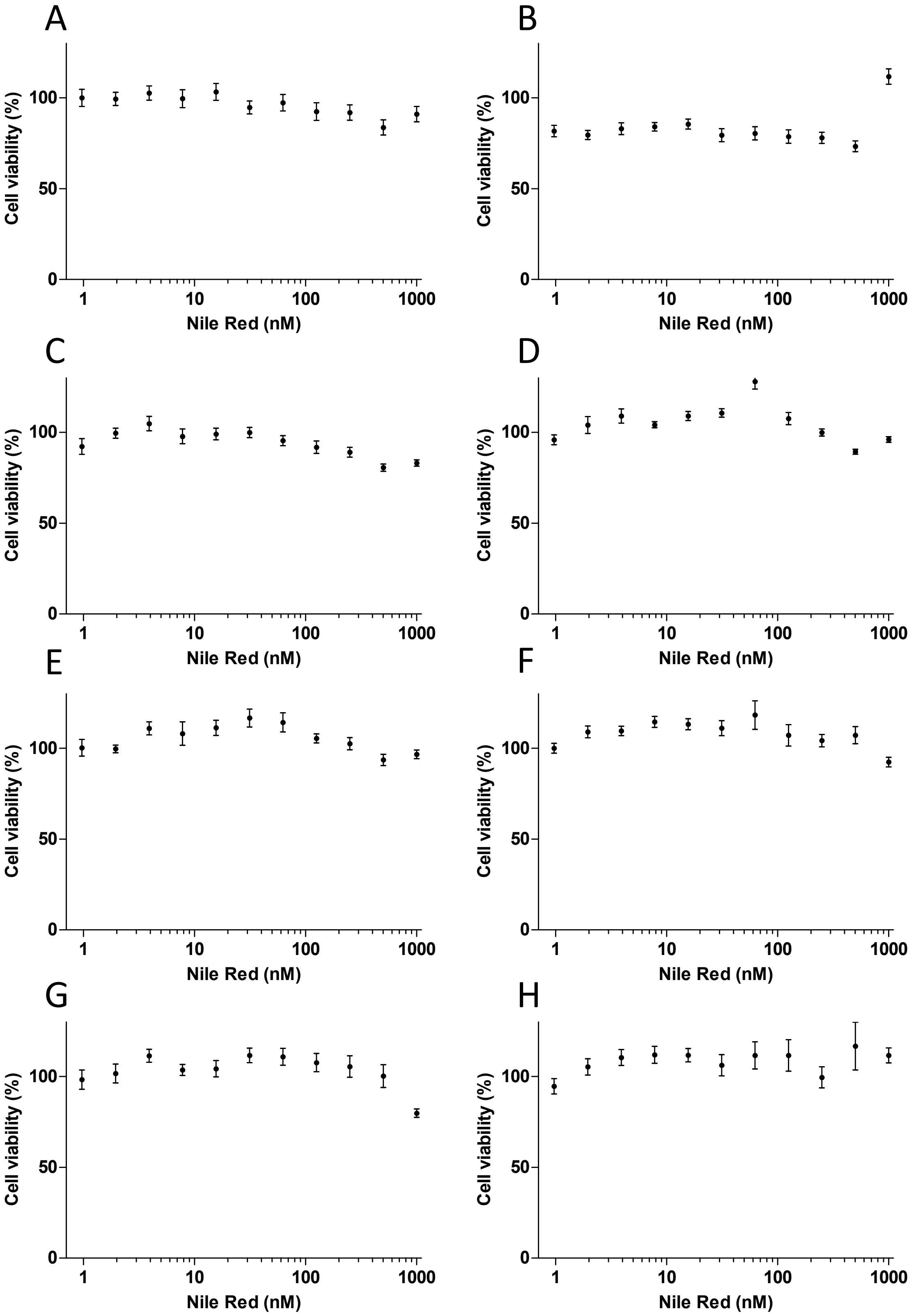

Figure S12c - ATP assay of Caco-2 cells following 24 hour incubation with aqueous Nile Red and each polydendron. A = aqueous Nile Red, EC_{50} 1.946. B =**G_2** Dendron:PEG_{16} 0:100, EC_{50} 2.855. C = **G_2** Dendron:PEG_{16}10:90, No EC_{50}. D = **G_2** Dendron:PEG_{16}25:75, No EC_{50}. E = **G_2** Dendron:PEG_{16}50:50, No EC_{50}. F = **G_2** Dendron:PEG_{16}75:25, No EC_{50}, G = **G_2** Dendron:PEG_{16}90:10, EC_{50} 2.848. H = **G_2** Dendron:PEG_{16}100:0, EC_{50} 0.1961.

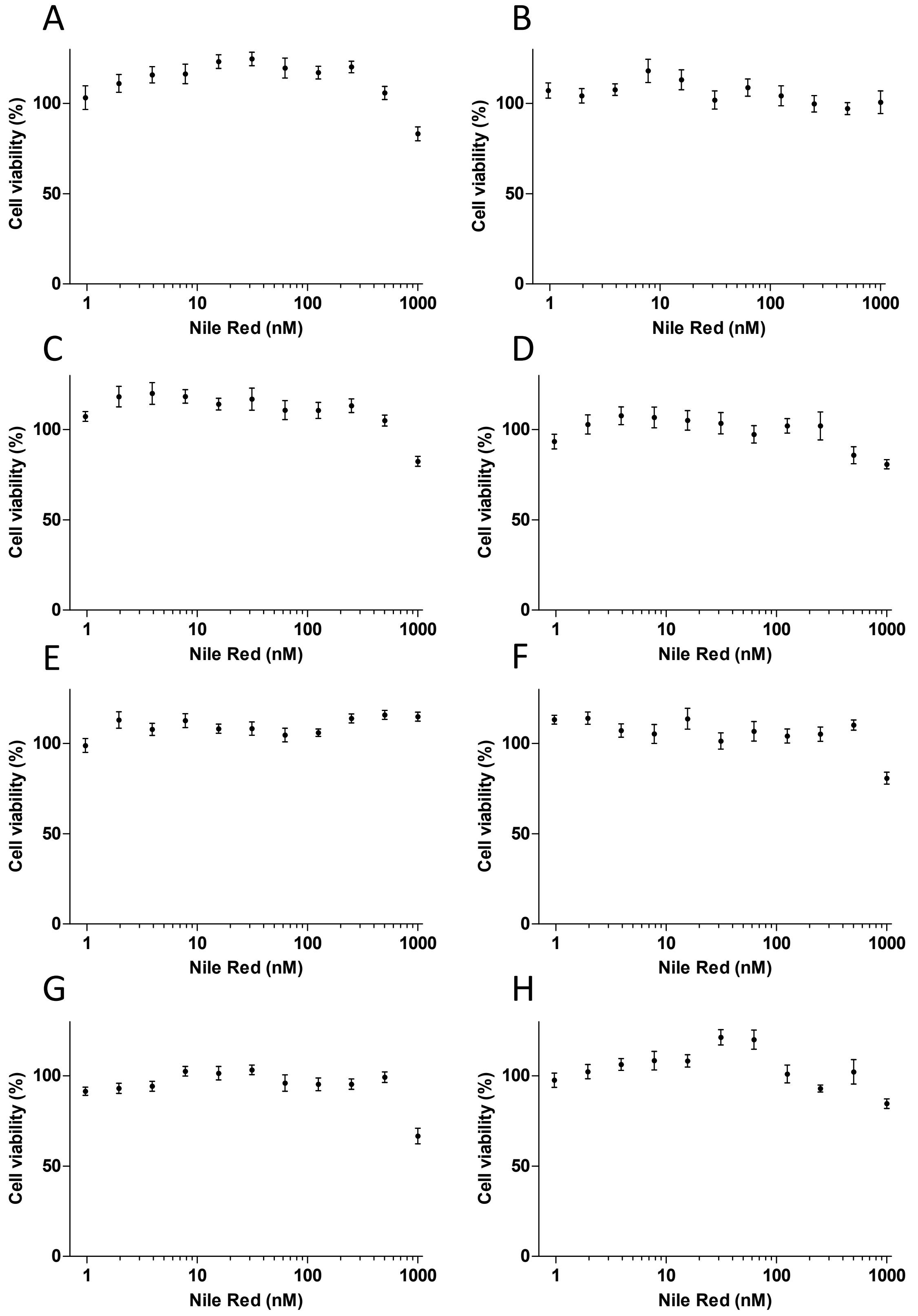

Figure S12d - ATP assay of Caco-2 cells following 120 hour incubation with aqueous Nile Red and each polydendron. A = aqueous Nile Red, No EC_{50}. B = $\mathbf{G_2}$ Dendron:PEG_{16} 0:100, No EC_{50}. C = $\mathbf{G_2}$ Dendron:PEG_{16} 10:90, EC_{50} 3.168. D = $\mathbf{G_2}$ Dendron:PEG_{16} 25:75, EC_{50} 2.565. E = $\mathbf{G_2}$ Dendron:PEG_{16} 50:50, No EC_{50}. F = $\mathbf{G_2}$ Dendron:PEG_{16} 75:25, EC_{50} 3.032, G = $\mathbf{G_2}$ Dendron:PEG_{16} 90:10, No EC_{50}. H = $\mathbf{G_2}$ Dendron:PEG_{16} 100:0, No EC_{50}.

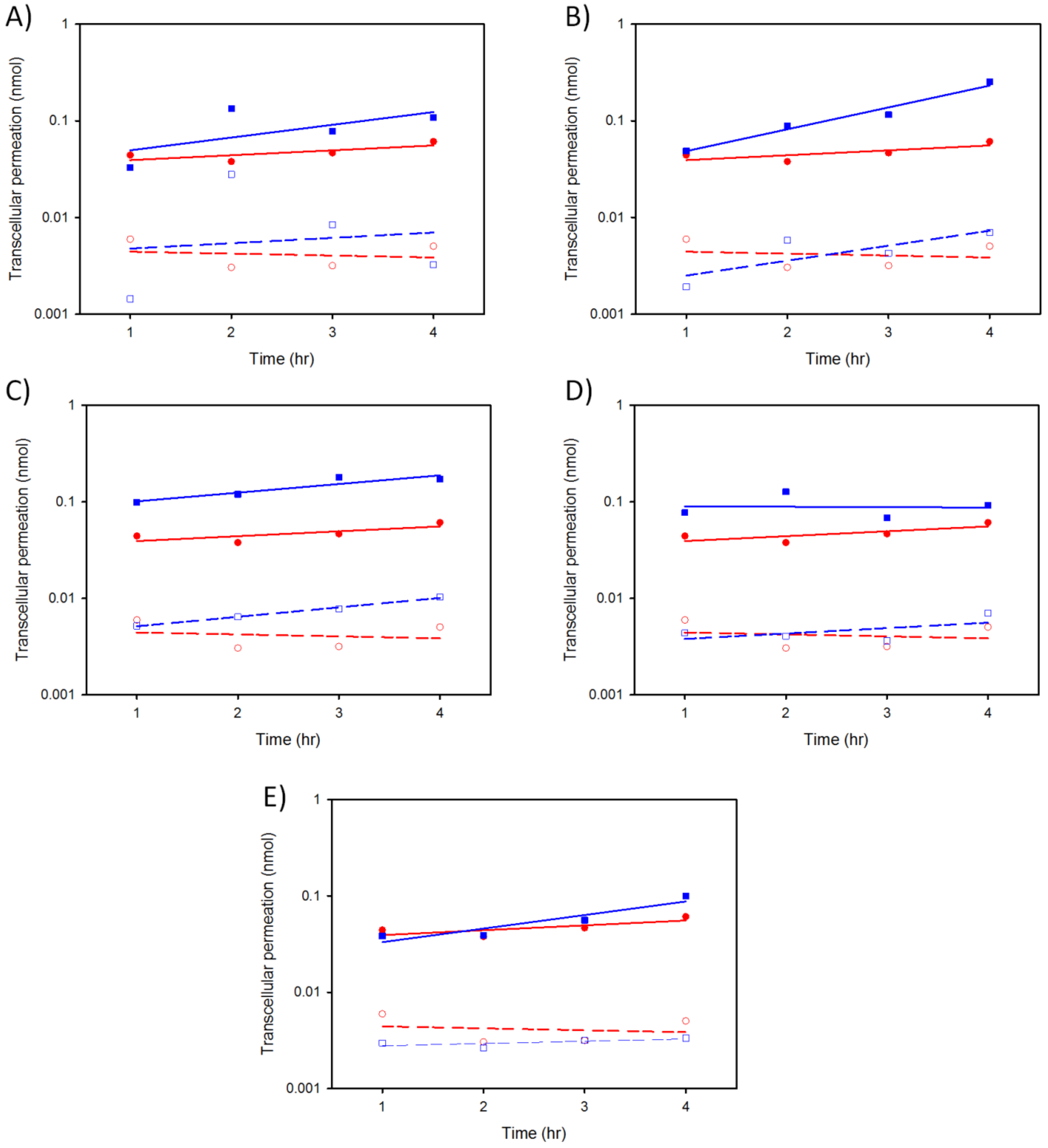

Figure S13 – Transcellular permeability across Caco-2 cell monolayers (4 hour time period) of polydendrons containing encapsulated Nile Red relative to an aqueous solution of Nile Red. $\mathbf{G_2}$ dendron:PEG_{16} variation A) 100:0; B) 75:25, C) 50:50; D) 25:75; E)0:100. Each graph indicates the permeation from A>B (closed squares) and B>A (open squares) for aqueous Nile Red (red data) and each polydendron (blue data). Data are given as the mean of experiments conducted in biological triplicate.

Materials

All starting materials, α-bromoisobutyryl bromide (98 %), 4-(dimethylamino)pyridine (≥ 99 %), triethylamine (≥ 99 %), hydroxypropyl methacrylate (97 %), ethylene glycol dimethacrylate (98 %) Cu(I)Cl (≥ 99 %), 2,2'-bipyridyl (≥99 %), poly(ethylene glycol) monomethyl ether (average M_n 750 $gmol^{-1}$) were purchased from Aldrich and used as received. Anhydrous methanol and Dowex® MarathonTM MSC (hydrogen form) ion exchange resin beads were purchased from Aldrich and used as received. All other solvents were analytical grade and purchased from Fisher. The $\mathbf{G_2}$ dendron, **1**, was synthesised as reported previously.[1]

Characterisation

Molecular weights and molecular weight distributions (*i.e.* dispersity, Đ) were measured using a Malvern Viscotek instrument equipped with a GPCmax VE2001 auto-sampler, two Viscotek T6000 columns (and a guard column), a refractive index (RI) detector VE3580 and a 270 Dual Detector (light scattering and viscometer) with a mobile phase of THF at 35°C and a flow rate of 1.0 $mLmin^{-1}$.

NMR spectra were recorded using a Bruker DPX-400 spectrometer operating at 400 MHz for ^{1}H NMR and 100 MHz for ^{13}C, in $CDCl_3$, D_2O or DMSO.

Dynamic light scattering (DLS) measurements were performed using a Malvern Zetasizer Nano ZS instrument (laser wavelength; 630 nm), ran at 25 °C.

Scanning electron microscopy (SEM) images of nanoparticles were obtained using a Hitachi S-4800 FE-SEM. The aqueous nanoparticle samples were dropped on a glass cover slide mounted on an aluminium stub with a carbon tab and left to dry over several hours or overnight. They were Au sputter coated at 20 mA for 2 min prior to imaging.

Fluorescence spectra were obtained on a PerkinElmer Luminescence spectrofluorophotometer LS55. Emission spectra for Nile red containing samples were recorded between 550 nm and 700 nm, exciting at 552 nm. The slit widths for emission and excitation were 5 nm and 10 nm, respectively, with a scan rate of 100 nm/min. Emission spectra for pyrene labelled samples were recorded between 330 nm and 500 nm, exciting at 335 nm. The slit widths for emission and excitation were 5 nm and 5 nm, respectively, with a scan rate of 100 nm/min.

Experimental Procedures

PEG$_{16}$ initiator 4 synthesis

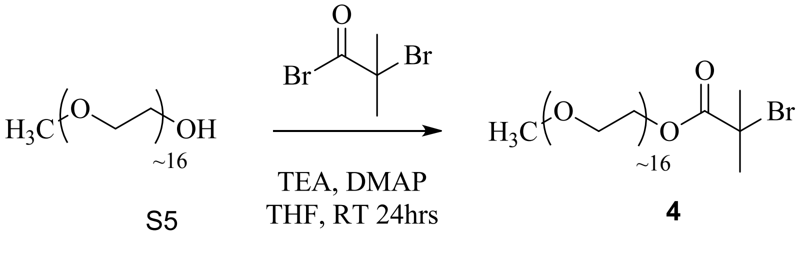

Figure S14 – Synthesis scheme for PEG$_{16}$ initiator **4**

Monomethoxypoly(ethylene glycol) (S5, Mw ≈ 750 g/mol) (23.0 g, 30.7 mmol) was dissolved in warm THF (~40 °C), and the reaction was degassed with dry N_2. DMAP (37.5 mg, 0.3 mmol) and TEA (7.48 mL, 53.7 mmol) were added and the reaction was cooled to 0 °C in an ice bath. α-bromoisobutyryl bromide (5.69 mL, 46.0 mmol) was added dropwise over 30 minutes and a white precipitate appeared immediately; the $Et_3NH^+Br^-$ salt. After 24 hours the precipitate was filtered, THF removed *in vacuo* and the resulting crude product was precipitated from acetone into petroleum ether (30-40 °C) twice to give **4** (72 %). ^{1}H NMR (400 MHz, D_2O) δ ppm 4.31 (m, 2H), 3.77 (m, 2H), 3.70-3.59 (m, 60H), 3.55 (m, 2H), 3.31 (s, 3H) and 1.89 (s, 6H). ^{13}C NMR (100 MHz, D_2O) δ ppm 174.0, 71.5, 70.4, 70.1, 70.0, 68.8, 58.6, 30.5.

Linear dendritic polymer synthesis with G_2 dendron initiator

In a typical experiment, $\mathbf{G_2}$ dendron initiator,**1,**(0.648 g, 0.69 mmol)and HPMA, **2**, (targeted DP_n = 50) (5.0 g, 34.7 mmol) were weighed into a round bottom flask. The flask was equipped with magnetic stirrer bar, sealed and degassed by bubbling with N_2 for 20 minutes and maintained under N_2 at30 °C. Anhydrous methanol was degassed separately and subsequently added to the monomer/initiator mixture via syringe to give a 50 wt% mixture with respect to the monomer. The catalytic system; Cu(I)Cl (0.069 g, 0.69 mmol) and 2,2’-bipyridyl (bpy) (0.217 g, 1.39 mmol), were added under a positive nitrogen flow in order to initiate the reaction. The polymerisations were stopped when conversions had reached over 98 % determined by ^{1}H NMR using the vinyl CH_2 peaks and protons of the polymer backbone. The polymerisation was stopped by diluting with a large excess of tetrahydrofuran (THF), which caused a colour change from dark brown to a bright green colour. The catalytic system was removed using Dowex® Marathon™ MSC (hydrogen form) ion exchange resin beads and basic alumina. The resulting polymer was isolated by precipitation from the minimum amount of acetone into cold hexane. The [initiator]:[CuCl]:[bpy] molar ratios in all polymerizations were 1:1:2.

G_2 polydendron synthesis

In a typical experiment, $\mathbf{G_2}$ dendron initiator, **1**,(0.648 g, 0.69 mmol)and HPMA, **2**, (targeted DP = 50) (5.0 g, 34.7 mmol) were weighed into a round bottom flask. EGDMA, **3**, (105 µl, 0.55 mmol) was added and the flask was equipped with magnetic stirrer bar, sealed and degassed by bubbling with N_2 for 20 minutes and maintained under N_2 at30 °C. Anhydrous methanol was degassed separately and subsequently added to the monomer/initiator/brancher mixture via syringe to give a 50 % v/v mixture with respect to the monomer. The catalytic system; Cu(I)Cl (0.069 g, 0.69 mmol) and 2,2’-bipyridyl (bpy) (0.217 g, 1.39 mmol), were added under a positive nitrogen flow in order to initiate the reaction. The polymerisations were stopped when conversions had reached over 98 % determined by ^{1}H NMR using the vinyl CH_2 peaks and protons of the polymer backbone. The polymerisation was stopped by diluting with a large excess of tetrahydrofuran (THF), which caused a colour change from dark brown to a bright green colour. The catalytic system was removed using Dowex® Marathon™ MSC (hydrogen form) ion exchange resin beads and basic alumina. The resulting polymer was isolated by precipitation from the minimum amount of THF into cold hexane. The [initiator]:[CuCl]:[bpy] molar ratios in all polymerizations were 1:1:2.

Mixed initiator branched polymerisations *via* ATRP

In a typical reaction, $\mathbf{G_2}$ dendron initiator (0.324 g, 0.35 mmol) and 2K PEG initiator (0.745 g, 0.35 mmol) (for a targeted ratio of $\mathbf{G_2}$ dendron:2000PEG of 50:50 mol%) were

weighed into a round bottom flask, followed by HPMA (5.0 g, 34.7 mmol, targeted DP=50). EGDMA (112 μL, 0.59 mmol, 0.85 mol%) was added and the flask was equipped with magnetic stirrer bar, sealed and degassed by bubbling with N_2 for 20 minutes and maintained under N_2 at30 °C. Anhydrous methanol was degassed separately and subsequently added to the monomer/initiator/brancher mixture *via* syringe to give a 50 % v/v mixture with respect to the monomer. The catalytic system; Cu(I)Cl (0.069 g, 0.69 mmol) and 2,2'-bipyridyl (bpy) (0.217 g, 1.39 mmol), were added under a positive nitrogen flow in order to initiate the reaction. The polymerisations were stopped when conversions had reached over 98 %. The polymerisations were stopped by diluting with a large excess of tetrahydrofuran (THF), which caused a colour change from dark brown to a bright green colour. The catalytic system was removed using Dowex® Marathon™ MSC (hydrogen form) ion exchange resin beads and basic alumina. The resulting polymer was isolated by precipitation from the minimum amount of THF into cold hexane. The [initiator]:[CuCl]:[bpy] molar ratios in all polymerizations were 1:1:2.

Kinetics experiments

Kinetics experiments were conducted at 30 °C. To determine the kinetic parameters of polymerisations samples (~0.1 mL) were taken at regular intervals and analysed by ^{1}H NMR and gel permeation chromatography (GPC). Approximately one third of each sample taken was diluted into 0.8 mL of deuterated DMSO (for ^{1}H NMR analysis) and the other two thirds was diluted into 4mL THF (for GPC analysis). Oxidation of Cu(I) to Cu(II) was observed by a colour change from brown to turquoise in DMSO and from brown to green in THF indicating termination of the reaction. Conversion of monomer to polymer was determined by ^{1}H NMR as previously discussed. The samples diluted in THF were prepared for GPC analysis by removal of the catalytic system with Dowex® Marathon™ MSC (hydrogen form) ion exchange resin beads, transferred into a pre-weighed vial, removal of THF from the polymer solution by evaporation to give dry polymer residue of a known weight. The dry polymer residues of known weights were then diluted with HPLC grade THF to give polymer solutions with concentrations around 5.0 mg/mL. These were analysed by triple detection GPC consisting of refractive index (RI), light scattering (LS) and viscometer detectors.

Aqueous nanoparticle formation

Polymers were dissolved in THF at various concentrations. Once fully dissolved, polymer in THF (0.1 or 1 mL at 5 or 10 mg/mL) was added quickly to a vial of water (5 or 10 mL) stirring at ambient temperature. The solvent was allowed to evaporate overnight in a fume cupboard to give a final concentration between 0.01 - 2 mg/mL polymer in water. By adjusting the starting concentration and the volume of water used, the size of the corresponding nanoparticles were controlled.

Encapsulation of guest molecules in nanoparticles

Polymers were dissolved in THF at various concentrations. The fluorescent dyes, Nile red and pyrene, were dissolved in THF to give a stock solution (0.1 mg/mL). The stock solution (50 μL, 0.1 mg/mL) was added to an empty vial and the THF was allowed to evaporate to leave 5 μg of dye. The polymer dissolved in THF (1 mL, 5 mg/mL) was added to the dry Nile red or pyrene to give a mixture containing 5 mg polymer, 5 μg dye dissolved in 1 mL of THF. This was then added quickly to a vial of water (5 mL) stirring at ambient temperature.

The solvent was allowed to evaporate overnight in a fume cupboard to give a final concentration of 1 mg/mL polymer and 1 μg/mL Nile red or pyrene (0.1 w/w%) in water.

Caco-2 Monolayer Permeation Studies

Materials

Dulbecco's Modified Eagles Medium (DMEM), Hanks buffered saline solution (HBSS), Trypsin-EDTA, bovine serum albumin (BSA), Nile red, 3-(4,5-Dimethylthiazol-2-yl)-2,5—diphenyltetrazolium bromide (MTT reagent), acetonitrile (ACN) and all general laboratory reagents were purchased from Sigma (Poole, UK). Foetal bovine serum (FBS) was purchased from Gibco (Paisley, UK). The CellTiter-Glo® Luminescent Cell Viability Assay kit was from Promega (UK). The 24-well HTS transwell plates were obtained from Corning (New York, USA). The 96-well black walled, flat bottomed plates were from Sterilin (Newport, UK).

Characterisation

Cell count and viability was determined using a Countess automated cell counter (Invitrogen).

Absorbance was read using a Tecan Genosis plate reader at 560 nm (Tecan Magellan, Austria).

Luminescence was then measured using a Tecan Genios plate reader (Tecan Magellan, Austria).

Experimental Procedures

Cytotoxicity studies (Nile red loaded *hyp*-polydendrons)

Caco-2 cells were seeded at a density of 1.0 x 10^4 cells / 100 μL in DMEM supplemented with 15 % FBS into each well of a 96 well plate (Nunclon, Denmark) and incubated at 37 °C and 5 % CO_2. Cells from 4 separate flasks of biological replicates of each cell type were used (N1-4) to improve statistical power. Media was then aspirated from column 1 and replaced with media containing each *hyp*-polydendron or aqueous Nile red solution at an equivalent 1 μM Nile red concentration then diluted 1:1 in media across the plate up to column 11. Column 12 served as a negative control and consisted of media and untreated cells. Following *hyp*-polydendron addition, the plates were incubated for 24 hours or 120 hours at 37 °C, 5 % CO_2 prior to assessment of cytotoxicity.

MTT assay - Following incubation of treated plates for 24 hours or 120 h, 20 μL of 5 mg/mL MTT reagent was added to each well and incubated for 2 hours. Subsequently, 100 μL MTT lysis buffer (50% N-N-Dimethylformamide in water containing 20% SDS, 2.5% glacial acetic acid and 2.5% hydrochloric acid, pH 4.7) was added to each well to lyse overnight at 37 °C, 5% CO_2. Following incubation the absorbance of each well was read using a Tecan Genosis plate reader at 560 nm (Tecan Magellan, Austria).

ATP assay - Following incubation of treated plates for 24 hours or 120 hours, cells were equilibrated to room temperature for approximately 30 minutes. All but 20 μL of media was removed from each well and 20 μL CellTiter-Glo® (Promega, UK) reagent was added. All

reagents were made fresh and in accordance with the manufacturer's instructions. Plates were put on an orbital shaker for 10 minutes to mix contents and allow for stabilisation of luminescence signal. Luminescence was then measured using a Tecan Genios plate reader (Tecan Magellan, Austria).

Caco-2 permeation studies (Nile red loaded *hyp*-polydendrons)

Transwells were seeded with 3.5 x 10^4 cells per well and propagated to a monolayer over a 21 day period, during which media in the apical and basolateral wells was changed every other day. Trans-epithelial electrical resistance (TEER) values were monitored until they were >800 Ω. 1 μM of Nile red *hyp*-polydendron or 1 μM aqueous Nile red was added to the apical chamber of 4 wells and the basolateral chamber of 4 wells to quantify transport in both Apical to Basolateral (A>B) and Basolateral to Apical (B>A) direction and sampled on an hourly basis over a 4 h time period. Apparent permeability coefficient (P_{app}) was then determined by the amount of compound transported over time using equation (1) below.

$$P_{app} = (dQ/dt)\ (1/AC_0) \qquad (1)$$

Where (dQ/dt) is the amount per time (nmol/sec), A is the surface area of the filter and C_0 is the starting concentration of the donor chamber (1 μM).

Aqueous Nile Red solution for cellular studies

An aqueous Nile Red solution was prepared in dimethyl sulfoxide (DMSO) at 1 mg/mL final concentration and used to spike either complete growth media or transport buffer. The resulting 1 μM final concentration Nile Red solution was subsequently used in cytotoxicity assays or for transcellular permeability assessment respectively. Transport buffer consisted of; Hanks buffered Saline Solution (HBSS), 25 mM4-(2-hydroxyethyl)-1-piperazineethanesulfonic acid (HEPES), and 1 mg/mL Bovine Serum Albumin (BSA), adjusted to pH 7.4.

Routine cell culture/cell maintenance

Caco-2 cells were purchased from American Type Culture Collection (ATCC, USA) and maintained in Dulbecco's Modified Eagles Medium (DMEM) supplemented with 15% filtered sterile foetal bovine serum. Cells were incubated at 37°C and 5% CO_2 and were routinely sub-cultured every 4 days when 90% confluent. Cell count and viability was determined using a Countess automated cell counter (Invitrogen).

Extraction and quantification of Nile Red

100 μl of each collected sample was mixed with 900 μl acetone, vortexed, sonicated for 6 minutes and centrifuged at 13300 rpm for 3 minutes. The supernatant was completely dried in a vacuum centrifuge at 30°C until the dry solid sample was left. This was reconstituted in 150 μl acetonitrile, transferred to a 96-well black walled, flat bottomed plate and measured for fluorescence intensity excitation wavelength 480 nm, emission wavelength 560 nm using a Tecan Genios plate reader (Tecan Magellan, Austria).

Statistical analysis

Statistical analysis of continuous data was assessed using simple linear regression. All statistical analyses were conducted in Stats Direct Statistical Software (Version 2.7.9) and each case, a P value lower that 0.05 was deemed to be statistically significant. Formal statistical analysis of cytotoxicity data (MTT and ATP assay) was not possible because none of the material exhibited cytotoxicity at the tested concentrations.

References

[1]F. L. Hatton, P. Chambon, T. O. McDonald, A. Owen and S. P. Rannard, *Chem. Sci.*, **2014**, *5*, 1844-1853.
